# Expression of VEGF and Semaphorin Genes Define Subgroups of Triple Negative Breast Cancer

**DOI:** 10.1371/journal.pone.0061788

**Published:** 2013-05-08

**Authors:** R. Joseph Bender, Feilim Mac Gabhann

**Affiliations:** Institute for Computational Medicine and Department of Biomedical Engineering, Johns Hopkins University, Baltimore, Maryland, United States of America; Kyushu Institute of Technology, Japan

## Abstract

Triple negative breast cancers (TNBC) are difficult to treat due to a lack of targets and heterogeneity. Inhibition of angiogenesis is a promising therapeutic strategy, but has had limited effectiveness so far in breast cancer. To quantify heterogeneity in angiogenesis-related gene expression in breast cancer, we focused on two families – VEGFs and semaphorins – that compete for neuropilin co-receptors on endothelial cells. We compiled microarray data for over 2,600 patient tumor samples and analyzed the expression of VEGF- and semaphorin-related ligands and receptors. We used principal component analysis to identify patterns of gene expression, and clustering to group samples according to these patterns. We used available survival data to determine whether these clusters had prognostic as well as therapeutic relevance. TNBC was highly associated with dysregulation of VEGF- and semaphorin-related genes; in particular, it appeared that expression of both VEGF and semaphorin genes were altered in a pro-angiogenesis direction. A pattern of high VEGFA expression with low expression of secreted semaphorins was associated with 60% of triple-negative breast tumors. While all TNBC groups demonstrated poor prognosis, this signature also correlated with lower 5-year survival rates in non-TNBC samples. A second TNBC pattern, including high VEGFC expression, was also identified. These pro-angiogenesis signatures may identify cancers that are more susceptible to VEGF inhibition.

## Introduction

Angiogenesis is the formation of new blood vessels from existing networks of capillaries. This blood vessel sprouting and remodeling, which is a normal part of organ growth and of adult physiology, can be co-opted to supply tumors by stimulating the growth of new branches from the host organ vasculature.

**Figure 1:**
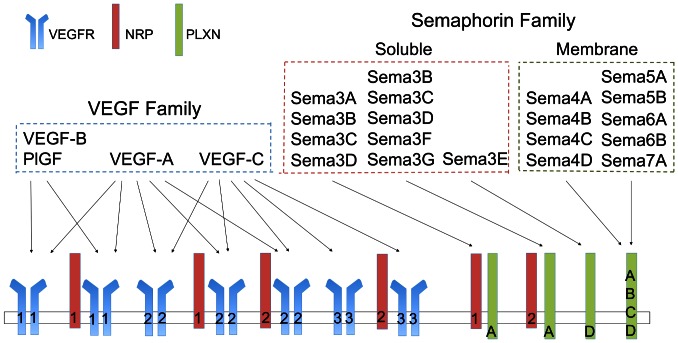
Ligand-Receptor interactions for the VEGF and Semaphorin families. VEGF ligands bind to and signal through three RTKs: VEGFR1, VEGFR2, and VEGFR3 (blue). Neuropilins are in red, with numbers to distinguish between neuropilin-1 and neuropilin-2. Semaphorin ligands bind to and signal through Plexins A-D (green). Many (but not all) members of the VEGF and Sema3 families use Neuropilin 1 or 2 as a co-receptor for binding to the canonical signaling receptors. This competition for Neuropilin is thought to represent one mechanism by which VEGF and Semaphorin ligands antagonize each other; in addition, the downstream signaling of VEGFRs and Plexins can have opposite function. Note that not all splice isoforms of VEGF-A, VEGF-B, and PlGF can bind to the receptors indicated, and that kinetic rates of binding vary among isoforms.

The vascular endothelial growth factor (VEGF) family (Figure 1) plays a large role in the regulation of angiogenesis. This family comprises five ligands (VEGFA, VEGFB, VEGFC, VEGFD, and PlGF) and three receptors (VEGFR1, VEGFR2, and VEGFR3). There are also two neuropilin co-receptors (NRP1 and NRP2). VEGFR2 signaling plays a prominent role in promoting angiogenesis, while VEGFR3 signaling promotes lymphangiogenesis [1], [2], 3.

Inhibition of angiogenesis, depriving tumors of nutrients by preventing the formation of a surrounding vasculature, has shown promise as a therapy for cancer. The VEGF-neutralizing antibody bevacizumab is currently approved for treatment of colorectal, lung, brain, and kidney cancers [4], [5], [6], [7]. Tyrosine kinase inhibitors such as sunitinib and sorafenib, which inhibit the kinase activity of VEGF receptors, are approved for use in kidney, pancreatic, stomach, and liver cancers [8], [9], [10], [11]. Angiogenesis inhibition has also been shown to have an effect on progression-free survival in breast cancer, but a lack of effect on overall survival has limited its use for this disease [12], [13]. Accelerated approval for bevacizumab in breast cancer was withdrawn in 2011 after 3 years. The limited effectiveness of these therapies – in particular the variability in efficacy between cancer types and even between individuals – necessitates a better understanding of the mechanisms through which VEGF signaling inhibitors act, and the environment in which they find themselves.

Varying responses to treatments among populations of breast cancer patients reflects the fact that breast cancer is a heterogeneous disease. Breast cancers are commonly divided into subgroups based on the expression of three cell surface receptors: estrogen receptor (ER), progesterone receptor (PR), and HER2. Tumors that are negative for all three of these receptors (“triple-negative”) tend to have poorer prognoses due to a more invasive phenotype and fewer treatment options [14]. A second, somewhat orthogonal classification defines breast tumors as basal or luminal; the major difference being which types of keratins are expressed, as determined by immunohistochemistry. A third classification uses gene expression signatures to group breast cancers into five intrinsic subtypes based on a subset of 50 genes (“PAM50”): a basal group and two luminal groups (luminal A and B), as well as normal-like and HER2-enriched groups. Luminal groups tend to be ER-positive while basal tends to be ER-negative [15]. Substantial overlap exists between triple negative breast cancers (TNBCs) and basal tumors [16]: a large proportion of TNBCs are basal, whereas a smaller proportion of non-TNBCs are basal [17]. TNBCs can be further subdivided into multiple different subtypes [18]. Angiogenesis inhibition is of particular interest in TNBCs, as the VEGF concentration and microvessel density are often higher in these tumors than in non-TNBC tumors [19], [20].

The effectiveness of therapies that target VEGF signaling may be modified by the presence of other ligands that can bind to the VEGF co-receptors neuropilin-1 and neuropilin-2. One such family of neuropilin-binding proteins are the class 3 semaphorins, which have been shown to have inhibitory effects on tumor progression and angiogenesis, possibly through competition with VEGF for binding to neuropilins [21], [22], [23], [24], [25], [26]. While class 3 semaphorins require neuropilins for binding and signaling though plexin receptors, other semaphorins (classes 4, 5, 6, and 7) bind to plexins directly [22]. Despite the lack of neuropilin binding by these semaphorins, they have been shown to affect VEGF signaling as well, either through direct interactions with VEGF receptors [27] or through modulation of downstream signaling pathways [28], [29]. These indirect VEGF-semaphorin interactions suggest that: (1) semaphorins may be novel anti-angiogenic therapeutic targets; and (2) semaphorins should be considered when determining patient subgroups that may be responsive to anti-VEGF therapies. Figure 1 shows a schematic of known VEGF-VEGFR-semaphorin-plexin interactions (the genes and their associated microarray probes are listed in Tables S2 and S3 in File S1).

In this study, we explore how gene expression of VEGF and semaphorin ligands and receptors is altered in a large number of breast tumors from many previously published microarray studies, totaling over 2,600 individuals. Our analysis identifies differences in VEGF and semaphorin ligand and receptor expression between triple negative tumors and other tumors, as well as differences among the subtypes of triple negative breast cancer.

## Results

### Expression Patterns Differentiating Normal and Tumor Tissue

**Figure 2:**
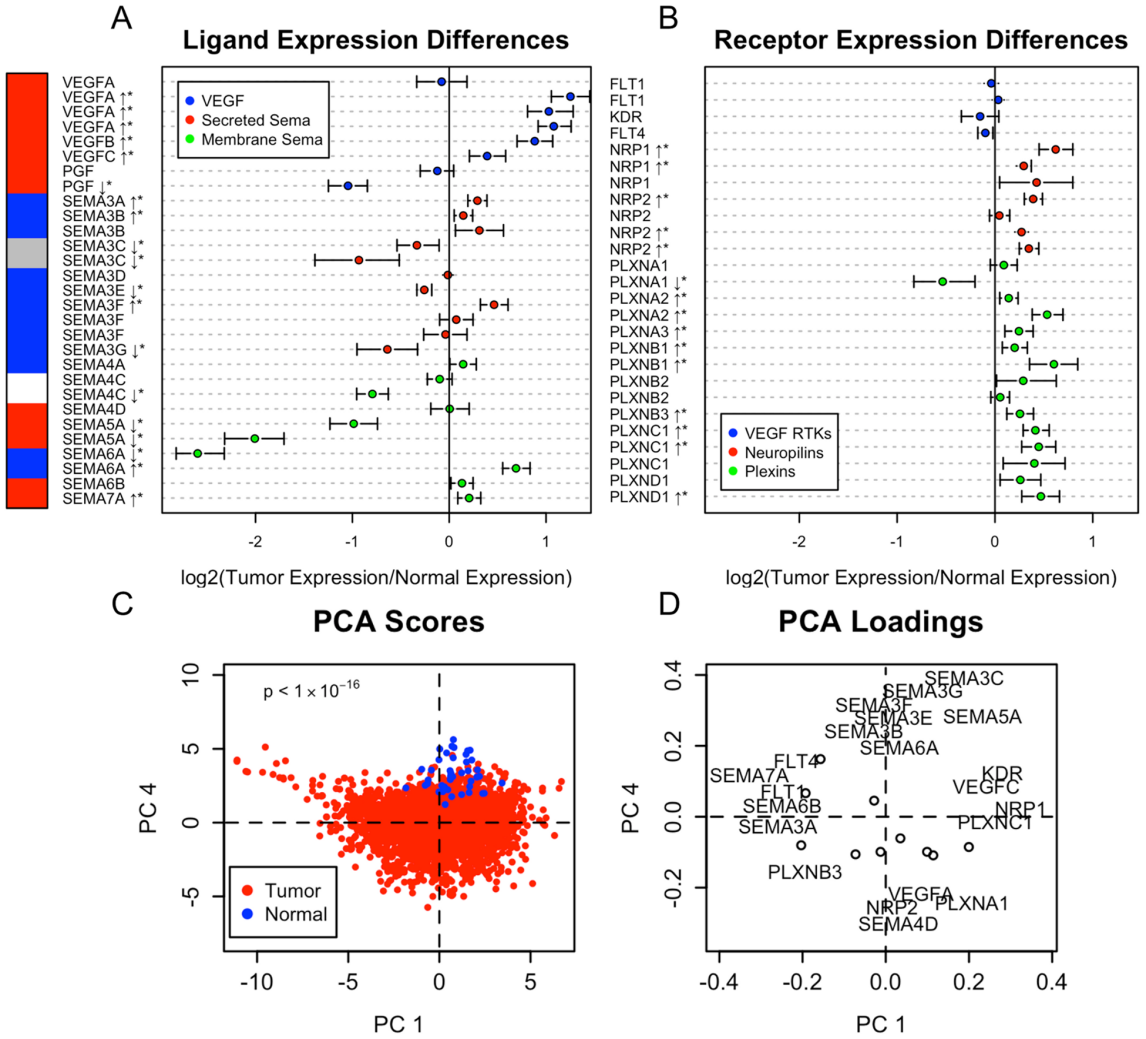
Differences in expression patterns of VEGF- and semaphorin-related genes between normal breast tissue (n = 42) and breast tumors (n = 2,656). **A–B**, Differences in mean expression with 99% confidence intervals as determined by the Wilcoxon rank sum test for (A) ligands and (B) receptors at the probe level. Ligands are marked with the following colors to denote known effect on angiogenesis: red for pro-angiogenic, blue for anti-angiogenic, gray for context-dependent (could be pro-or anti-angiogenic) and white for unknown. Genes for which expression is significantly altered in tumors are denoted by * (p<0.001) and the direction noted by an arrow. **C–D**, Principal component analysis shows separation of tumors from normal samples based on first and second principal component scores (C), with corresponding gene expression patterns given by the loadings for these components (D). Gene labels are only shown for genes whose loading vectors onto PC1 and PC4 exceed a magnitude of 0.23. Circles denote the loading of genes whose names do not appear.

The 2,656 tumor samples were compared to 42 normal samples to identify differentially expressed genes. Overall, 20 out of 29 of the ligand probe sets differed between tumor and normal tissues at a significance level of 0.01 (Figure 2A). All of the VEGF ligands were up-regulated in tumors except PlGF. PlGF had two probe sets on the U133A platform: one was down-regulated and the other did not differ significantly between normal and tumor tissues. Semaphorin ligand expression differences varied, with a mix of up- and down-regulation. Ligands in Figure 2A were annotated on the left axis with a color of red if they were known to have pro-angiogenic functions, blue for anti-angiogenic functions, gray if they had been shown to both promote and inhibit angiogenesis in different studies, or white if no data were available. The studies supporting these designations are listed in Table S2 in File S1 [3], [23], [27], [28], [29], 30,31,32,33,34,35,36,37,38,39,40,41,42,43,44. In general, class-3 semaphorins inhibit angiogenesis, while the other four classes have varying effects.

Although 20 out of the 26 receptor probe sets differed from normal expression at a significance level of 0.01 (Figure 2B), the overall magnitude of the fold change between tumor and normal tissue was less for receptors than for ligands: the mean absolute difference was 0.58 for ligands (mean of all points in Figure 2A) compared to 0.32 for receptors (mean of all points in Figure 2B). Most receptors that were differentially expressed were up-regulated, with the exception of FLT4 (VEGFR3) and PLXNA1.

We used principal component analysis (PCA) to determine patterns of covariation in gene expression between tumor and normal samples. Projection of the gene expression data onto the plane defined by principal components 1 and 4 (PC1, PC4) showed the best separation between tumor and normal samples (Figure 2C). Normal samples showed moderate values of PC1 and high values of PC4; tumor samples had a broader range of PC1 values and lower values of PC4. High PC1 scores were associated with high expression of VEGFC, KDR, NRP1, and PLXNC1, and low expression of SEMA3A, SEMA7A, FLT1, and FLT4. Low PC1 scores were associated with the opposite expression pattern. Low PC4 scores were associated with a pro-angiogenesis signature, a combination of high expression of VEGFA, SEMA4D, NRP2, and PLXNA1, and low expression of several secreted semaphorins: SEMA3B, SEMA3C, SEMA3E, SEMA3F, and SEMA3G. The advantage of the PCA-based approach for comparing tumor and normal samples over the differential expression analysis used in Figures 2A and 2B was that patterns of co-expression could be observed. For example, KDR expression by itself was not significantly altered between tumor and normal samples (Figure 2B), but co-expression of KDR with VEGFC, NRP1, and PLXNC1 was associated with tumors (Figure 2D).

### VEGF and Semaphorin Gene Expression are Differentially Regulated in Triple Negative Breast Cancer

To determine patterns in VEGF and semaphorin expression that may be important in distinguishing various breast cancer subgroups, we performed PCA on the expression measurements for the 31 VEGF- and semaphorin-related genes in the data set consisting of 2,656 tumors. We compared the scores obtained from PCA with commonly used clinical variables and found that the principal components had the most significant associations with triple negative status (Figure S1C in File S1), as indicated by the large logistic regression coefficients. Some significant associations were found between the principal components and lymph node status (Figure S1D in File S1) and tumor grade (Figure S1E in File S1), but the coefficients were much smaller than those for triple negative status. Tumor stage was not associated with the components at all (Figure S1F in File S1). Additionally, we noted that applying PCA to the VEGF-related gene subset alone failed to distinguish TNBC samples from receptor-positive samples as effectively as the combined data set (Figure S1A in File S1). Applying PCA to the semaphorin-related gene subset alone resulted in some significant associations with triple-negative status (Figure S1B in File S1). The NRP1 and NRP2 genes were included in both subsets. Together, this suggests that the (indirect) interactions between the VEGFs and the semaphorins lead to different neuropilin-regulated signaling activities in TNBC tumors compared to receptor-positive tumors.

**Figure 3:**
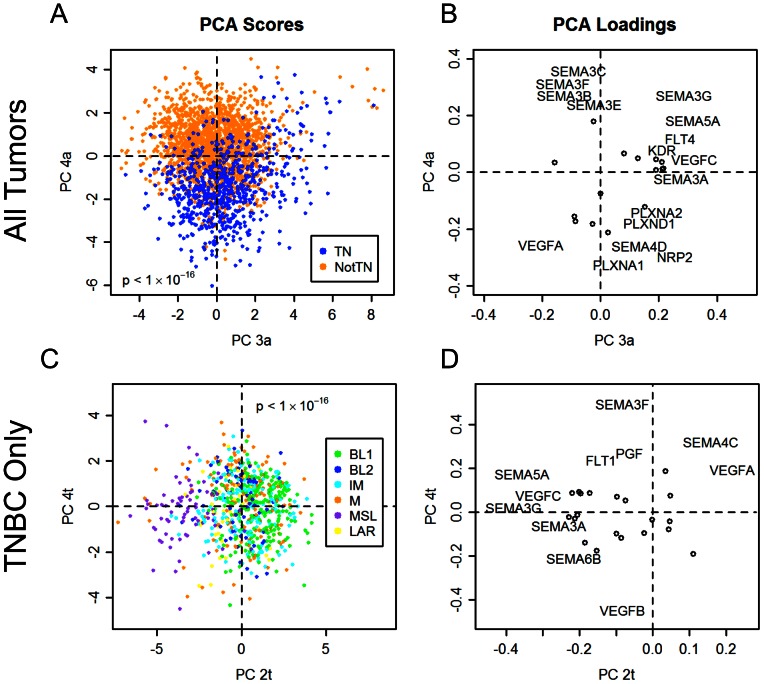
Triple negative breast cancers and the mesenchymal stem-like (MSL) subtype of triple negative breast cancers are associated with increased pro-angiogenic gene expression and decreased anti-angiogenic gene expression. **A–B**, Principal component analysis (PCA) scores (A) and loadings (B) for VEGF- and semaphorin-related genes in all tumors. Gene names are only shown for probes whose loadings on the two principal components exceeded a radius of 0.2 from the origin. Circles denote genes whose names do not appear. Triple-negative (TN) samples project to lower values of tumor PC4a, corresponding to high VEGFA and low SEMA3 expression. **C–D**, PCA scores (C) and loadings (D) for VEGF- and semaphorin-related genes in only the TN samples. The MSL subtype projected to low values of TNBC PC2t, corresponding to up-regulation of VEGFC, SEMA3G, and SEMA5A and down-regulation of VEGFA.

When applying PCA to the combined VEGF and semaphorin data set, the projection of the data onto the fourth principal component (PC4a, “a” to denote the all-tumor data set) provided the highest degree of separation between TNBC samples and the rest of the tumors (Figure 3A), with low values of PC4a corresponding to TNBC samples. A group of tumors also scored highly on PC3a. A relatively large proportion of these were TNBC samples that did not score low on PC4a. PC1a had a slight association with TNBC status but only in samples that scored low on PC4a, while PC2a did not appear to have any association with TNBC status. PC4a also was significantly associated with the basal subtype (as defined by the PAM50 gene signature classifier described previously [45]) (Figure S2 in File S1), consistent with the similarities between TNBCs and the basal subtype.

Low PC4a scores were associated with a pro-angiogenic signature consisting of high expression of VEGFA, SEMA4D, NRP2, and PLXNA1 and low expression of SEMA3B, SEMA3C, SEMA3E, SEMA3F, and SEMA3G. High PC3a scores were associated with high expression of VEGFC, SEMA3A, SEMA3G, SEMA5A, KDR, and FLT4, and low expression of VEGFA (Figure 3B). Clustering based on just the PC3a and PC4a scores resulted in two groups of tumors with higher amounts of TNBCs (clusters 1 and 3, respectively, in Figure S3 in File S1). In addition to the established roles of VEGFA and VEGFC as promoters of angiogenesis, published experimental data has shown that other genes associated with low PC4a and high PC3a, SEMA4D and SEMA5A, also have pro-angiogenic function [29], [39], [40] (Table S2 in File S1). Interestingly, three of the ligands with reduced expression in high PC3a and low PC4a samples, SEMA3B, SEMA3F, and SEMA3G, had both anti-angiogenic and tumor suppressor functions [23], [30], [31], [36], [37], [38] (Table S2). The role of SEMA3C in angiogenesis has not been well-defined, but like other class-3 semaphorins, it binds to neuropilin receptors. Thus, it may impair signaling by members of the VEGF family by competing for neuropilin.

Examining the correlations between PC4a scores and all genes whose expression was measured on the U133A platform revealed that the ESR1 gene, which encodes ER, had the second highest correlation with PC4a of all genes (Table S7 in File S1). Other transcription factors associated with ER, such as GATA3 and FOXA1, had high correlations as well. This indicated that the association between PC4a score and TN status may arise primarily because of an association with ER, as opposed to PR or HER2. ER, PR, and HER2 did not appear in the list of the most correlated genes with PC3a scores (Table S6 in File S1).

### The MSL Subtype Differs Significantly from Other TNBC Subtypes

Next we examined VEGF and semaphorin expression in TNBC samples assigned to the TNBC subtypes discovered in Lehmann et al [18]. PCA of VEGF and semaphorin expression for only the TNBC samples revealed that of all of the subtypes, the mesenchymal stem-like (MSL) subtype was most distinguishable from the others (Figure 3C). The MSL subtype projected to low values of the second principal component (PC2t, “t” to denote the TNBC-only data set). The gene expression pattern corresponding to low PC2t included low expression of VEGFA and high expression of VEGFC, SEMA5A, and SEMA3G (Figure 3D). This was similar to the PC3a from the analysis of all tumors in the previous section (Figure 3B), except that the signs were reversed (Figure S4 in File S1, also see Table 1 for a comparison of expression signatures across all principal component analyses in this study). There was substantial overlap between the triple negative tumors that had high PC3a scores in Figure 3A and the tumors that had low PC2t scores in Figure 3C, indicating that the MSL subtype could likely be distinguished even in the PCA of all of the tumors.

**Table 1:** VEGF/Sema gene PCA loading patterns.

	All Tumors (PC1a–4a)	TNBC (PC1t–4t)	TCGA-microarray (PC1m–4m)	TCGA-RNA-Seq (PC1r–4r)
**PC1**	**High** VEGFC, KDR, NRP1	**High** FLT1, FLT4, SEMA3A, 6B, 7A	**High** VEGFC, KDR, PGF, NRP1	
	**Low** FLT1, FLT4, SEMA3A, 7A	**Low** KDR, NRP1	**Low** SEMA4F, 4G	**Low** VEGFC, FLT1, KDR, FLT4, NRP1
**PC2**	**High** PLXNB1, PLXNB2	**High** VEGFA	**High** VEGFA, PLXNA1	**High** SEMA3B, 3F, PLXNB1
	**Low** SEMA4D	**Low** VEGFC, KDR, NRP1,SEMA3G, 5A	**Low** SEMA3B, 3C, 3F, PLXNB1	
**PC3**	**High** VEGFC, KDR, FLT4,SEMA3A, 5A	**High** PLXNB1, B2, B3		**High** VEGFA, PLXNA1, SEMA7A
		**Low** SEMA4D, PLXNC1	**Low** PLXNA3, B3	**Low** SEMA3C
**PC4**	**High** SEMA3B, 3C, 3E, 3F	**High** SEMA3F	**High** SEMA5B, 6A, 6B	**High** SEMA3A
	**Low** VEGFA, SEMA4D, PLXNA1	**Low** VEGFB	**Low** NRP2, PLXNC1, SEMA3A,4D, 7A	**Low** VEGFA, PGF, FLT1, KDR, FLT4

The genes with the largest magnitude loadings are shown for each component across the 4 different principal component analyses performed in this study. All of the expression patterns listed here correspond to samples with high scores for the particular component.

The median expression of VEGFA, VEGFC, SEMA5A, and SEMA3G in the MSL subtype was closer to the median of receptor-positive tumors than to that of TNBC samples. In the case of VEGFA, SEMA5A, and SEMA3G, the expression levels in the MSL subtype were closer to those of normal tissues than the tumor average. On the other hand, VEGFC was expressed at higher levels on average than in any of the other groups (Figure S7 in File S1), indicating that this subgroup may be susceptible to angiogenesis inhibitors that target VEGFC instead of VEGFA.

### Consensus Clustering Defines VEGF- and Semaphorin-based Tumor Subtypes

**Figure 4:**
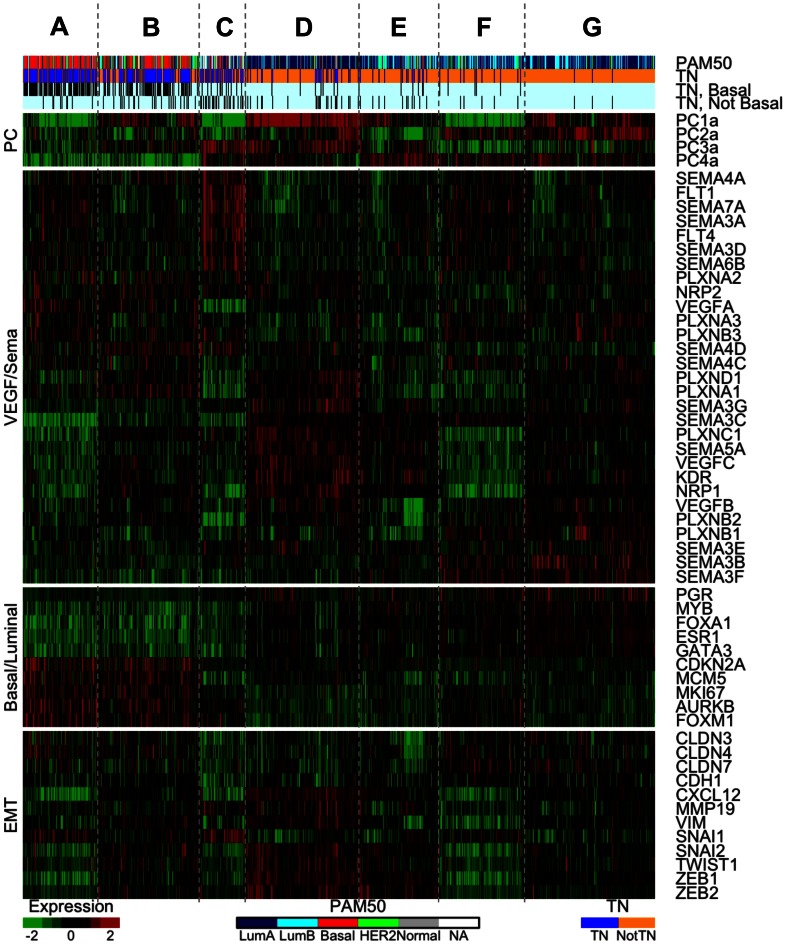
Heatmap of the 7 VEGF/Sema-based tumor clusters. Samples are ordered across the columns by cluster membership as determined by consensus *K*-means clustering. The clusters are ordered by TN content, with cluster A on the left having the highest percentage of TNBCs. The VEGF/Sema-based clusters are able to differentiate the basal intrinsic subtype from the other intrinsic (PAM50) subtypes. As previously noted, the basal subtype is strongly associated with triple negative tumors (third and fourth bars: black for basal TNBCs and non-basal TNBCs, respectively). Other breast cancer-related genes have expression patterns that align with the VEGF/Sema-based clusters.

We used consensus *K*-means clustering to determine VEGF- and semaphorin-related subtypes independent of any other classifications. This differed from the PCA-based clusters in Figure 3 in that all gene expression variation was analyzed here to determine the natural clusters that arise in VEGF- and semaphorin-related gene expression. Consensus clustering revealed 7 clusters (labeled A–G) for “all tumors” data (Figure S8 in File S1), and 5 clusters (labeled J–N) for “TNBC-only” data (Figure S9 in File S1). The gene expression for the seven tumor clusters is illustrated in the heatmap in Figure 4, with the clusters arranged by the number of TNBCs present in decreasing order.

#### High VEGFA-expressing clusters

The first two clusters (A and B) in Figure 4 possessed the pro-angiogenic PC4a gene expression signature noted in Figure 3, namely high VEGFA expression and low expression of SEMA3B/3C/3F. Cluster A is distinguished from cluster B by higher expression of FLT1 (VEGFR1), FLT4 (VEGFR3), and several semaphorins (including SEMA3A), and lower expression of VEGFC, KDR (VEGFR2), and NRP1. Both clusters have high percentages of TNBCs (78% and 66%, respectively). Using the intrinsic classifier (PAM50, Figure 4), these two clusters were found to contain most of the basal subtype tumors. As expected, most of the TN tumors were basal as well (rows 3 and 4 of Figure 4 compare the overlap between the TN and basal subtypes). Both of these clusters had low expression of the genes encoding for ER and PR (ESR1 and PGR) and of some of their associated transcription factors (GATA3, FOXA1, MYB), and high expression of proliferation-related genes (the basal/luminal panel in Figure 4), consistent with the basal subtype.

#### High VEGFR1/VEGFR3-expressing cluster

Cluster C in Figure 4 had high expression of FLT1 (VEGFR1), FLT4 (VEGFR3), SEMA3A, and some other semaphorins, with low expression of VEGFA, VEGFC, KDR (VEGFR2), NRP1, SEMA3C, PLXNA1, and PLXND1. This cluster also had a relatively high percentage of TNBCs (43%), but with a lower amount of basal subtype tumors than the high VEGFA-expressing clusters. This indicated that although many TNBCs had the pro-angiogenic PC4a signature, it was not strictly required for a tumor to be triple-negative. This cluster had low expression of the claudin genes CLDN3, CLDN4, and CLDN7, raising the possibility that tumors in this cluster were members of the claudin-low subtype, a group of breast tumors known for their invasive, mesenchymal-like behavior.

#### High VEGFC-expressing cluster

Cluster D in Figure 4 had high expression of a group of genes including VEGFC, KDR (VEGFR2), NRP1, and SEMA5A. This corresponded to the alternative pro-angiogenic TNBC PC2t signature noted in Figure 3. This cluster had some TNBCs (16%). Significant overlap was noted between this cluster and the luminal A subtype of the PAM50 intrinsic classifier; 67% of cluster D tumors were luminal A, representing 30% of all luminal A tumors. Cluster D was notable in that it had the highest expression of transcription factors implicated in the epithelial-to-mesenchymal transition (EMT), including SNAI2, TWIST1, ZEB1, and ZEB2 (the panel labeled “EMT” in Figure 4). This could indicate a role for VEGFC-mediated signaling in tumors undergoing an EMT.

#### High SEMA3-expressing clusters

Clusters F and G in Figure 4 had the anti-angiogenic high PC4a signature described previously: high expression of the anti-angiogenic semaphorins SEMA3B, SEMA3E, and SEMA3F, with low expression of VEGFA. These clusters had the lowest number of TNBCs and were mostly luminal A or B when classified into the PAM50 intrinsic subtypes. The pattern of expression of luminal markers and proliferation-related genes was opposite to that noted for the high VEGFA-expressing clusters: expression of ESR1, PGR, and associated transcription factors was high while expression of proliferation-related genes was low.

### Consensus Clustering Defines VEGF- and Semaphorin-based TNBC Subtypes

**Figure 5:**
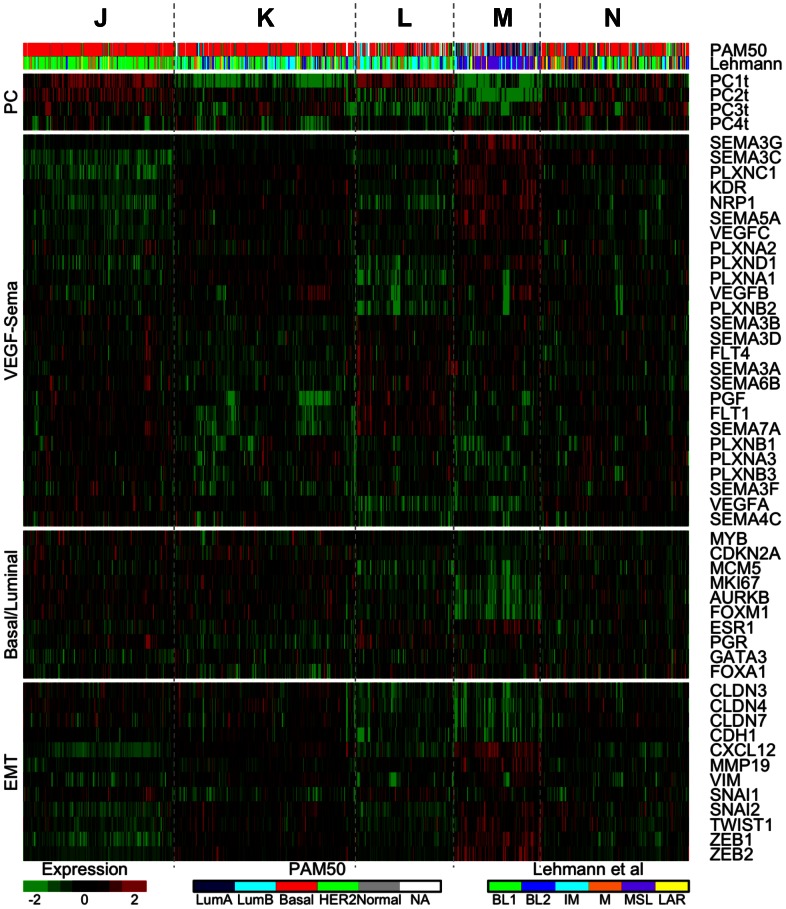
Heatmap of the 5 VEGF/Sema-based TNBC clusters. Consensus *K*-means clusters of only the TNBC data are arranged according to the ordering from Figure 4, in that the TNBCs that appeared in tumor cluster A generally now appear in TNBC cluster J (although the correspondence is not perfect) and so on for the other clusters. The VEGF/Sema-based clusters are able to differentiate the MSL subtype from Lehmann et al. (cluster M) from the other TNBC subtypes. Claudin-low subtype-related patterns of gene expression in the panel labeled “EMT” were associated with cluster M, the MSL-enriched cluster.

The 5 TNBC clusters, denoted J–N (Figure 5), were ordered as closely as possible to the tumor clusters in Figure 4. Thus the majority of samples in cluster A of Figure 4 fall into cluster J of Figure 5, and so on. The relationship is not perfect; many samples are differentially classified between the two cluster analyses.

The 5 TNBC clusters had similar expression patterns to the clusters described above for all tumors. TNBC clusters J and K had higher VEGFA expression on average, and as with clusters A and B, were differentiated by the pattern of high FLT1, FLT4, SEMA3A in cluster J and high KDR, NRP1 in cluster K. Clusters L and M had lower VEGFA expression, with cluster L expressing high levels of PGF, FLT1, FLT4, and SEMA3A, and cluster M expressing high levels of VEGFC, KDR, and NRP1. Cluster D had low PC2t scores from Figure 3C/D. The fifth cluster was not particularly distinguishable from the other TNBC clusters. Notably, all the clusters except cluster M were heavily populated by tumors of the basal PAM50 intrinsic subtype. This was seen for clusters A and B in the all-tumor data set (Figure 4), but not for clusters C and E. This is further evidence for the association between the basal subtype and TNBC; basal tumors comprised a small minority of clusters C and E in Figure 4, but this minority became the majority in Figure 5 when only the TN tumors were considered.

The TNBC subtypes found in Lehmann et al [18] had some associations with the VEGF−/Sema-based clusters found here. Of tumors in the MSL subtype, 81% were found in cluster M, comprising 65% of the tumors in that cluster. This corresponds to the PCA results that demonstrated a strong association of PC2t with the MSL subtype. Most of the other subtypes were evenly distributed across the clusters, with the exception of the basal-like 1 (BL1) subtype, which comprised 66% of cluster J.

Patterns of expression for other genes when sorted in the order of the TNBC clusters were less apparent in Figure 5 than in Figure 4, with cluster M showing the most significant regulation. Growth-associated genes such as FOXM1, AURKB, and MKI67 were strongly down-regulated in this cluster while the EMT-associated transcription factors SNAI2, TWIST1, ZEB1, and ZEB2 were up-regulated. Notably, the claudin genes CLDN3, CLDN4, and CLDN7 were down-regulated the most in this cluster, consistent with the observation that the MSL subtype and claudin-low subtype are closely related [19].

### Validation Using TCGA Data

**Figure 6:**
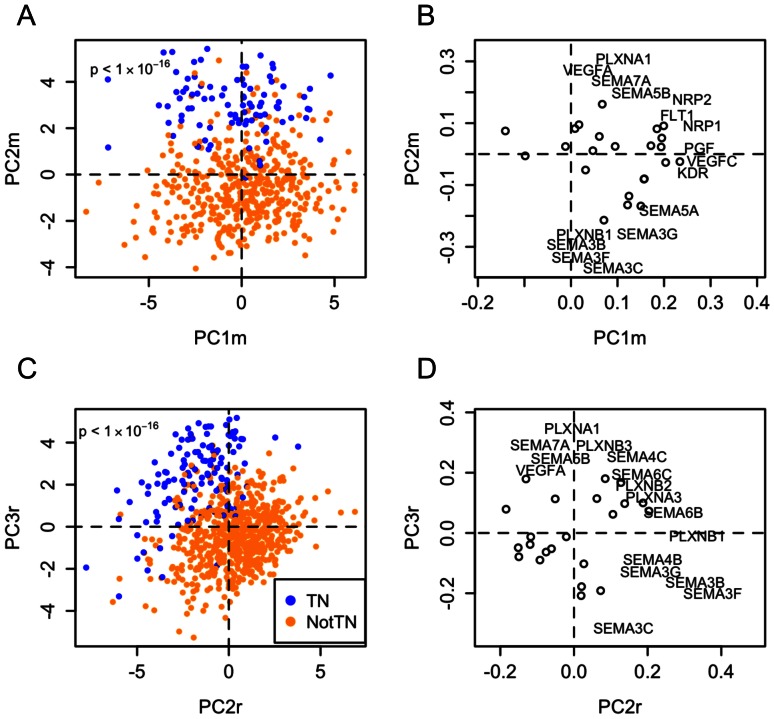
Validation of VEGF- and Semaphorin-related gene expression differences between triple negative and non-TN breast cancers using TCGA data. **A–B**, Principal component analysis of TCGA microarray data set consisting of 537 breast tumors. **C–D**, Principal component analysis of TCGA RNA-Seq data set consisting of 750 tumors. Both data sets were processed to gene-level measurements (rather than probe-level) prior to downloading. Tumors were classified as triple negative based on gene expression data for the ESR1, PGR, and ERBB2 genes as described in the *Methods*, all of which had clear bimodal distributions.

Using two TCGA data sets consisting of 537 tumors quantified using a different microarray platform (Figures 6A and 6B) and 750 tumors quantified using an RNA-Seq platform (Figures 6C and 6D), we showed that the same patterns of gene expression that distinguish TNBCs from other tumors could be found in other patients using different technologies. PCA scores between the two platforms used in the TCGA datasets had strong correlations (Figure S5 in File S1). Patterns of gene expression associated with the 2,656-tumor dataset were found in both TCGA datasets (Figure S6 in File S1), including the low PC4a gene expression signature of high VEGFA and low SEMA3B, SEMA3C, SEMA3F, and PLXNB1. Some additional genes were altered consistently in the two validation data sets as well: SEMA5B, SEMA7A, and PLXNA1. All three of these had similar expression patterns to that of VEGFA (Table 1 and Figure S10 in File S1).

### Survival Analysis of Clusters

**Figure 7:**
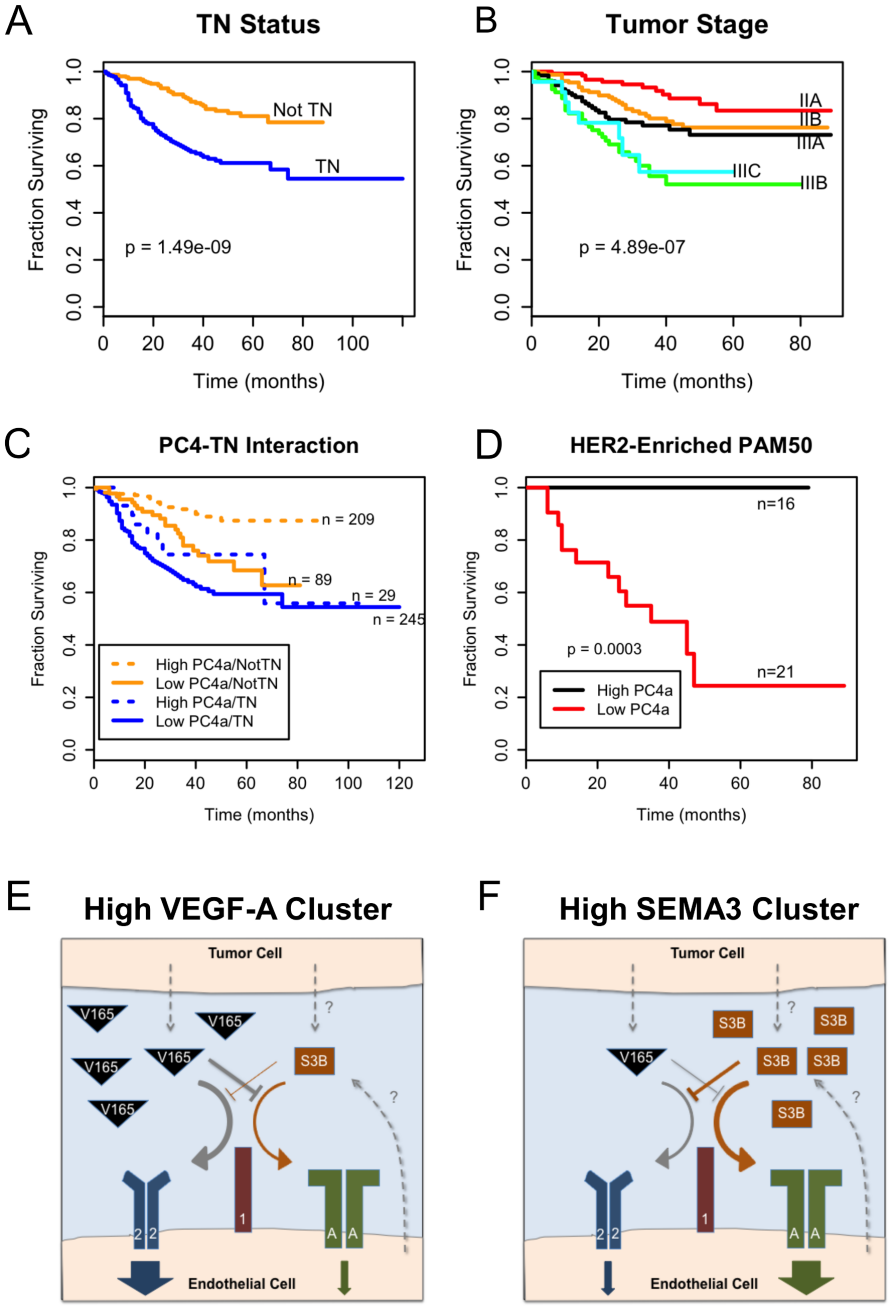
Angiogenesis gene expression subgroups correlate with survival. **A–D**, Survival curves for tumor samples that had available survival data. A log-rank test was used to determine p-values. Triple negative (TN) status was associated with worse prognosis for 572 patients with survival data (A). Higher tumor stage correlated with worse prognosis in 508 patients with available stage and survival data (B). Non-TNBC patients with low PC4a scores had poor prognoses similar to the TNBC patients (C). Patients in the HER2-enriched PAM50 subtype had significantly poorer prognoses if they also had low PC4a scores (D). **E–F**, Schematic of VEGF/semaphorin competition in the tumor microenvironment. The gene expression patterns of the different subgroups of TNBC and other cancers suggest different regulation of pro- and anti-angiogenesis pathways. The case with high expression of VEGFA and low SEMA3B, which corresponds to the high PC4a group, results in increased signaling through VEGF receptors such as VEGFR2 (blue). Most TNBC fit this profile, although many non-TNBC did also and these showed decreased 5-year survival similar to TNBC (C). Lower expression of VEGFA with high SEMA3B, corresponding to the low PC4a group, results in reduced signaling through VEGF receptors and more signaling through semaphorin receptors such as PLXNA1 (green). Note that these schematics only consider receptor expression on endothelial cells; signaling by plexins or VEGFRs on tumor cells may also play a significant role.

We performed Kaplan Meier survival analysis on the tumors to determine the impact of the PCA-derived clusters on patient prognosis. Triple negative status and increasing stage of the tumor were both correlated with poorer prognoses as expected (Figures 7A and 7B). Multivariate survival analysis using a Cox proportional hazards model showed that tumor stage, lymph node status, PC3a score, and PC4a score were all independent prognostic factors (Table 2). Interestingly, triple negative status, which was clearly correlated with poor survival, was not significant in the multivariate model (p = 0.06). The likely reason for this is that TN status is also highly correlated with PC4a scores. There were more non-TNBC samples with low PC4a scores (n = 89) than TNBC samples with high PC4a scores (n = 29), possibly resulting in a stronger survival effect from PC4a score than from TN status (Figure 7C). We also examined the interaction of ESR1 expression with PC4a; both low ESR1 expression and low PC4a scores were significantly associated with poor prognoses (Figure S12 in File S1). Interestingly, PC4a score was significantly associated with survival in a subgroup consisting of tumors with high ESR1 expression. Although ER+ tumors are already associated with effective therapies, combination of existing therapies with angiogenesis inhibition may provide additional benefits for these low PC4a, ER+ tumors.

**Table 2:** Survival analysis.

	Univariate		Multivariate	
Parameter	HR	*p*-value	HR	*p*-value
TN	2.83	4.9*10^−7^	1.72	0.06
Grade 3 vs. 1 or 2	1.57	**0.03**	0.87	0.55
Stage III vs. IIA or IIB	2.42	1.5*10^−5^	1.58	**0.05**
Age >50	1.03	0.89	1.02	0.91
Lymph node positivevs. negative	3.16	4.2*10^−5^	2.17	**0.02**
PC1a>median PC1a	0.86	0.69	0.87	0.73
PC2a>median PC2a	0.69	0.08	0.91	0.66
PC3a>median PC3a	1.81	**0.003**	1.75	**0.01**
PC4a>median PC4a	0.364	7.3*10^−6^	0.52	**0.02**
PC5a>median PC5a	1.22	0.33	1.21	0.36
PC6a>median PC6a	1.61	**0.02**	1.30	0.21

A Cox proportional hazard model demonstrated that the third and fourth principal components had significant effects on survival of patients, even when accounting for clinical variables.

Survival analysis by cluster (Figure S11A in File S1) showed that tumor clusters F and G had significantly better outcomes than the rest of the clusters. These were the only clusters that had both low PC3a scores and high PC4a scores (both anti-angiogenic signatures), reinforcing the prognostic value of these two principal components. The five TNBC clusters found here did not have significantly different prognoses; instead the survival curves of the clusters shared the same poor prognosis characteristic of TNBCs (Figure S11B in File S1). Despite the lack of variability, the differences in patterns of VEGF and semaphorin gene expression may indicate different growth factor dependencies. For example, VEGFC-targeting therapies may be more effective in cluster M, while the rest may benefit more from VEGFA-targeting therapies.

Within each PAM50 subtype, ESR1 was not significant (Figure S13 in File S1) while PC4a score was significantly associated with survival only in the HER2-enriched subtype (Figure 7D). The lack of association between survival and PC4a in the basal and luminal PAM50 groups (Figure S13 in File S1) is not unexpected since PC4a is somewhat consistent within these groups (low in basal, high in luminal, as shown in the heatmap in Figure 4). The prognostic effects of PC4a score in the HER2-enriched subtype could indicate that a low-PC4a subgroup of patients treated with HER2-targeting therapy may benefit from the addition of an anti-angiogenic drug to their treatment. Overall the association of survival and PC4a score in particular subtypes may aid in selecting patients where anti-angiogenic therapy would provide the greatest benefit.

## Discussion

Just as individuals have distinct genomic and gene expression profiles, so too the tumors of each individual are distinct. Understanding and quantifying this variability and individuality is crucial for the development and targeting of therapeutics for diseases as complex and heterogeneous as cancer. Triple negative breast cancers (TNBCs), in particular, are a diverse and difficult-to-treat set of tumors defined primarily by molecular targets for treatment that they do not express, rather than targets that they do express. Angiogenesis, a blood vessel morphogenesis process underpinning the growth and metastasis of most tumors, is a possible common target for TNBCs, and vascular endothelial growth factor (VEGF) has been targeted in breast cancer as a key regulator of angiogenesis. However, this has succeeded only for a subset of breast cancer patients, and thus understanding which subsets of patients may be responsive to this treatment is desirable. This requires data from a large number of patients, and we used one type of patient population data, gene expression microarrays, to quantify changes in VEGF and semaphorin expression to define relevant patient subgroups.

A high proportion of the genes considered here were significantly different between normal breast tissue and breast tumors (34/55 probe sets with p<0.001). This high rate of significance may be an indicator that these genes are heavily regulated by the genomic changes that occur in tumors. Alternatively, the low number of normal samples (n = 42) relative to tumor samples (n = 2,656) may result in an unrealistic estimate for significance. It is important to note that the range/variability in tumor expression is very high compared to the normal samples, as indicated by the standard deviations of each group (Table S4 in File S1). Although the mean values of expression for tumors and normal tissues may differ, the range of tumor expression often overlaps the range of normal expression. This makes individual genes poor biomarkers; however, they can be combined to identify tumor subgroups that correlate with differences in tumor characteristics.

The overall expression changes were consistent with previously reported breast cancer data of these genes at the mRNA and protein level. The increased expression of VEGFA in TNBCs compared to non-TNBCs was consistent with previous work that found an approximately 3-fold increase of VEGF as measured by ELISA of intra-tumoral samples from 679 patients [46]. VEGFR2 (KDR) was previously found to be significantly associated with TNBC in a panel of tissue microarrays from 564 patients [46]. This is consistent with our results, which showed a relatively high loading of KDR on the principal component associated with VEGFC, NRP1, and PLXND1, which had the second highest association with triple-negative (TN) status in the all-tumor data set. The increased expression of VEGFC in TNBC samples found here has also been demonstrated in IHC of breast cancer sections, where VEGFC stained positively in TNBCs significantly more often than in non-TNBCs [47]. Studies examining the amount of semaphorins and plexins expressed in breast cancer patients by TNBC and non-TNBC subgroups are not available, but there are some reports comparing their expression in normal breast and tumor tissue. SEMA3A, SEMA3B, SEMA3F, PLXNA1, and PLXNA3 were all shown by IHC to decrease as tumors progressed, while NRP1 increased and NRP2 stayed the same [48]. Another study showed the same pattern for PLXNA3 expression, while also showing that SEMA4F expression increased as tumors progressed [49].

Using multiple clustering and analysis algorithms, we have revealed patterns of gene expression associated with the triple-negative subtype of breast cancer that may indicate a higher collective pro-angiogenesis activity (Figure 4, tumor clusters A–C). In addition to up-regulation of the well-known angiogenic growth factor VEGFA in clusters A and B, several anti-angiogenic semaphorins were down-regulated. SEMA3B and SEMA3F have both anti-tumorigenic and anti-angiogenic properties [23], [30], [31], [36], [37], [50], [51]. SEMA3C, whose role in angiogenesis is less well-understood [32], [33], was also consistently down-regulated in tumors with high expression of VEGFA. Given that class-3 semaphorins compete with VEGF for binding to neuropilin co-receptors, this overall pattern of gene expression may enhance VEGF signaling in three ways: directly by increasing the amount of VEGFA; indirectly by reducing the amount of competitive inhibition for neuropilin; and decreasing anti-angiogenic Sema-Plexin signaling. The survival analysis in Figure 7C demonstrates the significance of this high-VEGF/low-Sema3 signature; patients with this signature have similar poor prognoses regardless of their triple negative status. The activity of angiogenesis in the TNBC-enriched cluster C from Figure 4 is less clear: VEGFA is down-regulated, while several semaphorins with both pro-angiogenic (SEMA6B and SEMA7A) and anti-angiogenic (SEMA3A and SEMA4A) effects are up-regulated.

What do these different gene-expression subgroups mean for treatment? We would hypothesize that high-VEGFA-expressing tumors would be more vulnerable to anti-VEGF treatment. Clinical trial results for the anti-VEGF drug bevacizumab have thus far not shown an increased efficacy in triple-negative subtypes [12], [52], [53], [54], [55] (Table S5 in File S1); instead, similar improvements have been seen in both triple negative and hormone receptor positive cancers (all cases were HER2-negative). However, we note that the high-VEGFA, low-Sema3 pattern (clusters A and B in Figure 4) makes up only 69% of all TNBC samples, and that 12% of non-TNBC samples can be classified as having a similar gene expression profile, possibly confounding this analysis.

Other clusters found in the tumor data may be less susceptible to inhibition of angiogenesis. The clusters with high expression of class-3 semaphorins would likely not benefit from this type of therapy because class-3 semaphorins function as endogenous inhibitors of angiogenesis. These tumors would be expected to be less aggressive; survival curves for patients with high class-3 semaphorin expression have the best prognoses (high PC4a in Figure 7C). The gene expression pattern consisting of high expression of VEGFC, PlGF, NRP1, and PLXND1 and low class-3 semaphorin expression (PC3a in Figure 3A/B) is also likely to be pro-angiogenic. However, it would not be expected to benefit from an anti-VEGFA therapy; instead, a different target would be needed to inhibit angiogenesis. This is supported by a report that low IHC staining of VEGFC and NRP1 is associated with improved progression-free survival in patients receiving bevacizumab, while the level of VEGFA was not associated with changes in progression-free survival [56].

The TNBC subtypes previously identified [18] demonstrated similar expression of VEGF- and semaphorin-related genes with the exception of the mesenchymal stem-like subtype. This subtype was noted for its enrichment of genes involved with migration and growth factor pathways, including KDR [18]. Here, we found a cluster of angiogenesis-related genes with increased expression in the MSL subtype, including VEGFC and KDR (TNBC cluster M in Figure 5, corresponding to tumor cluster D in Figure 4). Notably, however, VEGFA expression was decreased, indicating that although angiogenesis may occur in tumors of this subtype, VEGFA-targeted therapies are not likely to be successful inhibitors. In the analysis of all tumors, this VEGFC-dominated signature (tumor cluster D) was present in 18.5% of tumors. This cluster had a low proportion of triple negative tumors, raising the possibility that the MSL subtype may not just be a small subgroup within TNBCs, but a therapeutically relevant subgroup of breast cancers as a whole.

The concordance of the VEGF−/Sema-based clusters that we found here with expression patterns of genes associated with the basal/luminal distinction and EMT suggests that different breast cancer subtypes utilize the VEGF and semaphorin signaling pathways in consistently different ways. In particular, basal tumors with high expression of growth-associated genes such as MKI67 and AURKB tend to have higher levels of VEGFA, presumably to provide the rapidly proliferating cells with sufficient vasculature. On the other hand, tumors with low expression of growth-associated genes but high expression of EMT-associated transcription factors such as SNAI2 and TWIST1 have low VEGFA expression and high VEGFC expression. The lymphangiogenic VEGFC may facilitate invasion by allowing tumor cells to travel through the lymphatics, a commonly used route of metastasis in breast cancer [47]. This highlights the usefulness of this study not just in targeting anti-angiogenic therapies, but in understanding tumor biology as well.

One limitation of using gene expression microarrays on tumor samples taken from biopsies or surgeries is that the samples are heterogeneous. Along with the tumor cells they also contain stromal cells, including endothelial cells, fibroblasts, and immune cells. The expression of most of the ligands considered here can be assumed to be predominantly attributable to expression in the tumor cells, but for receptor expression the analysis is less straightforward. This is particularly true for receptors whose primary function of interest is on a cell type making up a small percentage of the total, e.g. endothelial cells. Their expression may be up-regulated in those cells but down-regulated in the more numerous cell type, resulting in detection of no or opposite change in expression in the microarray measurement of the heterogeneous sample. Immunohistochemistry can address this issue by measuring the cell-type-specific protein expression. For example, studies in a wide range of breast tumors have shown that NRP1 and NRP2 are both expressed on almost all endothelial cells, but very rarely on breast tumor cells [57], [58]. Conversely, PLXNB1 has been shown to be expressed on the surface of tumor cells, but less so on neighboring endothelial cells [59]. Thus, differences in expression of NRP1 and NRP2 measured by microarray can be assumed to be primarily due to endothelial cells, and differences in PLXNB1 due to tumor cells. Laser capture microdissection or other sorting methods could also resolve cell type differences by isolating specific cell types prior to analyzing gene expression. Methods such as these will be particularly useful in determining the relative amount of VEGF signaling taking place in tumor and endothelial cells.

We have yet to determine whether the VEGF- and Semaphorin-based clusters found here are recapitulated in gene expression data for breast cancer cell lines. Extensive work has been done to characterize the subtypes found in these cell lines and the differential susceptibility of the cell lines to various therapeutics [60], [61]. Many aspects of VEGF and semaphorin signaling depend on other cells in the tumor microenvironment, in particular endothelial cells and tumor stromal cells, and an analysis of cell lines could aid in determining which differentially expressed ligands in the present study arise due to tumor cells and which are due to stromal cells; as well as insight into whether observed receptor expression variation is due to tumor cells or tumor-associated endothelial cells.

We analyzed survival data in part to assess whether the VEGF- and Semaphorin-based clusters were associated with prognosis of breast cancer patients. The high correlation of VEGF- and Semaphorin-related gene expression with existing prognostic indicators such as TN status confounds the analysis and makes it impossible to determine from this data why some patients have poorer prognoses. However, we used a multivariate Cox proportional hazards model and Kaplan-Meier plots to demonstrate that a subgroup of ER+ tumors with the pro-angiogenic PC4a signature had poorer prognosis. Thus, the pro-angiogenic PC4a signature may have a role in severity of the disease, independent of ER or TN status. To determine the actual significance of the VEGF- and Semaphorin-based groups found here, experimental models of breast tumors are needed. Tumor xenografts in immunocompromised mice could be used to measure the growth and invasion of tumors of the various subtypes. This type of experimental model provides the advantage of allowing for other processes that contribute to cancer progression other than tumor cell growth, including angiogenesis. Targeted VEGF inhibitors and inhibitors of VEGF-pathway receptors could be administered to show whether the VEGF−/Semaphorin-based signature found here is truly relevant in tumorigenesis.

Computational models of VEGF and Semaphorin ligand-receptor interactions will be useful in unraveling the effects of the expression changes found here. The large number of proteins involved, combined with the complexity of their interactions, will make it necessary to use models to understand the overall effect of the expression patterns on signaling through VEGF receptors. Models of VEGF signaling [62], [63], [64] can be extended to include the Semaphorins found to be relevant in the current study. These models will enable prediction of patients expected to respond to existing therapies and can suggest effective therapeutic targets.

## Methods

### Data Sets

Published human breast cancer gene expression data sets were collated based on the following criteria: the tumors had to be untreated, primary tumors, and the gene expression had to be analyzed using the Affymetrix GeneChip® Human Genome U133A platform. Of the 98 data sets returned by searching for human breast tumors on the U133A platform in the GEO database, 22 met the criteria of being untreated and primary as of April 13, 2012. If available, the following data were also collected: ER, PR, and HER2 immunohistochemistry (IHC), lymph node status, age at diagnosis, tumor stage, and tumor grade. The breast cancer data sets [53], [65], [66], [67], [68], [69], [70], [71], [72], [73], [74], [75], [76], [77], [78], [79], [80], [81], [82], [83] (Table S1 in File S1) were compiled into one expression data set and normalized using the *justRMA* function in the *affy Bioconductor* package of the R statistical software environment. Some samples were removed prior to normalization: 30 samples in GSE20194 were replicates, 47 samples in GSE5847 were stromal cells isolated by laser capture microdissection, and 20 samples in GSE5847 had received neoadjuvant chemotherapy prior to surgery when the sample was taken. Many of the samples in different data sets were found to be from the same patients; samples were removed so that each patient was represented in the final data set only once. After removal of samples, the data set consisted of 2,656 individual tumor samples and 42 normal samples. When multiple probe sets corresponded to a single gene, only the probe set with the highest variance across all samples was used to represent expression of the gene.

The two TCGA data sets used for validation were current as of April 25, 2012. One data set consisted of 537 tumor samples analyzed on the Agilent G4502A microarray platform, while the other was an RNA-Seq data set consisting of 750 tumor samples analyzed on the Illumina HiSeq 2000 system. There were 481 patients overlapping between these two TCGA data sets. This replication was allowed in order to show similar results using different gene expression measurement technologies. Positive and negative status for the receptors was assigned based on IHC if available, otherwise based on the gene expression measurements for the ESR1, PGR, and ERBB2 genes.

### Assignment of TN Status and TN Subtypes

The assignment of triple negative status and subtype was made based on the gene expression levels of ESR1, PGR, and ERBB2 when IHC data were not available, as previously described by others [18]. A Gaussian distribution was fit based on expression of the three receptors for IHC TN samples, and another distribution was fit for IHC receptor-positive samples. Samples with no IHC data were classified by computing the probability of being TN based on the two density functions derived from samples with IHC data. Comparison of IHC data to expression-based assignments has demonstrated that misclassification of samples is rare (<3.6%) [18].

Subtypes of the triple negative classification were assigned by calculating subtype centroids based on the classification used previously by others [15]: expression of ∼2000 genes was used to compute centroids of each of the six subtypes based on 193 tumor samples. The classifier derived from this was tested using leave-one-out cross validation and classified 171 of the 193 samples correctly, for an accuracy of 88.6%. Testing the classifier trained with all 193 samples resulted in correct classification of 187 samples, for an accuracy of 96.9%. This classifier was used to determine the subtypes of the remaining 582 triple negative samples.

### PAM50 Intrinsic Subtypes

A previously used classifier for breast cancer involves the use of 50 genes to place tumors into one of five categories: basal, luminal A, luminal B, HER2-like, and normal-like. The method for classifying a new sample is to take the Spearman correlation coefficient of the expression of the 50 genes in the sample with each of the five class centroids. The class whose correlation coefficient is the highest is the class to which the sample belongs, unless all correlation coefficients are less than 0.1, in which case the sample is unclassified [84]. It should be noted that no genes in the PAM50 classifier overlap with the VEGF- and semaphorin-related genes that we consider here; thus when we compare VEGF- and semaphorin-based clusters, we are considering two completely independent methods of classification.

### Differential Expression

Genes for VEGF and semaphorin ligands and receptors that were significantly different between two groups (e.g. tumor vs. normal, receptor-positive vs. triple negative, etc.) were determined by the Wilcoxon rank sum test. This was carried out using the *wilcox.test* function in R.

### Principal Component Analysis

Principal component analysis (PCA) was used to reduce the dimensionality of the data sets from the 31 VEGF- and semaphorin-related genes under consideration to a smaller number of components that can reproduce most of the variability in the data. The components are linear combinations of the expression of the genes, and capture patterns of co-expression. The *prcomp* function in R was used to perform PCA. The columns of the *x* matrix returned by this function corresponded to the scores, while the columns of the *rotation* matrix corresponded to the gene loadings. For 2-D score plots where colors were used to show different groups of samples, the statistical significance of differences in PCA scores between the groups was determined using multivariate analysis of variance. The p-values were determined by comparing the Wilk’s lambda statistic to a chi-squared distribution.

### Logistic Rregression

For triple-negative status, lymph node status, tumor stage, tumor grade, age at diagnosis, and tumor size, logistic regression models were fit based on the scores of the first eight principal components using the R function *glm*.

### Survival Analysis

The R package *survival* was used to perform survival analysis on tumor samples for which survival and clinical variables were available. A log-rank test was used to assess univariate significance of factors. A Cox proportional hazards model was used for multivariate analysis of all factors.

### Cluster Analysis

*K*-means clustering was performed on the 2656-sample data set consisting of all of the breast tumors, as well as the 775-sample data set consisting of all triple negative tumors. The R function *kmeans* was used for clustering. To ensure that the algorithm converged to the global minimum instead of a local minimum, clustering was performed 50 times and the solution with the lowest within-class sum of squares was used to determine the cluster membership of each sample.

Consensus *K*-means clustering was used to assess the stability of the clusters. This consisted of performing the clustering algorithm 100 times on different subsets of the data set, and then computing the fraction of iterations in which any pair of samples were found in the same cluster. At each iteration, the sample subsets were determined by taking a random sample without replacement whose size was 80% of the data set. The consensus matrix is a visual representation of the fraction of iterations in which any pair of samples co-clustered. The cumulative distribution of the consensus matrix across all possible sample pairs was used to determine the number of clusters. The appropriate number of clusters was the cluster number at which no further increases in the area under the cumulative distribution curve occurred. Typically, the relative change in area is close to zero above a certain value of K. For example, in the all-tumor data set and the TNBC-only data set, values of K greater than and equal to 5 resulted in low relative area changes (Figures S6B and S7B in File S1). To select the appropriate number of clusters from these cases, the consensus matrices were investigated to determine which cluster number resulted in the most off-diagonal white space (Figures S6C-F and S7C-F in File S1) [84].

### Visualization of Data

Gene expression differences between tumor and normal samples were plotted in Figure 2A and B as the log of the ratio of the two means. Error bars corresponded to the 99% confidence interval of the log ratio derived from the Wilcoxon rank sum test. The range of gene expression across groups was shown in boxplots with the extreme ends of the boxes corresponding to the 25^th^ and 75^th^ percentile of the data and the line inside the box corresponding to median. The whiskers extended to the furthest point outside of the boxes that still fell within 1.5 times the interquartile range from the nearest end of the box, where the interquartile range was the difference between the 75^th^ and 25^th^ percentiles.

Heatmaps of gene expression data were generated in R using the *image* function. Data were scaled (zero-mean, unit-variance) and assigned colors, with red corresponding to high expression and green corresponding to low expression. Ordering of genes in the heatmaps was performed using the *hclust* function in R with the complete-linkage agglomeration method. When dendrograms were used, they were generated using the *plot.dendrogram* function in R.

## Supporting Information

File S1 Supporting information for this study. This file contains Tables S1–S7, which list the datasets and genes analyzed in this study, basic statistics on the gene expression measurements, clinical trial results with anti-angiogenic agents, and genes correlated with PC3a and PC4a. It also contains Figures S1–S13, which contain information on the relationship of principal components to breast cancer subgroups, relationships between the different principal component analyses performed on various datasets, and additional details on *K*-means clustering; plus heatmaps of the TCGA datasets and survival analyses of several of the breast cancer subgroups considered in this study.
(PDF)
