# Supplementary material for: Expression of VEGF and Semaphorin Genes Define Subgroups of Triple Negative Breast Cancer
Source: PLoS One. 2013 May 8;8(5):e61788. doi: 10.1371/journal.pone.0061788 (PMC3648524; doi:10.1371/journal.pone.0061788)
Supplement: File S1 — Supporting information for this study. This file contains Tables S1–S7, which list the datasets and genes analyzed in this study, basic statistics on the gene expression measurements, clinical trial results with anti-angiogenic agents, and genes correlated with PC3a and PC4a. It also contains Figures S1–S13, which contain information on the relationship of principal components to breast cancer subgroups, relationships between the different principal component analyses performed on various datasets, and additional details on K-means clustering; plus heatmaps of the TCGA datasets and survival analyses of several of the breast cancer subgroups considered in this study. (PDF) [file pone.0061788.s001.pdf]

Supplemental material for:

Expression of VEGF and Semaphorin genes define subgroups of triple negative breast cancer

R. Joseph Bender and Feilim Mac Gabhann

Institute for Computational Medicine and Department of Biomedical Engineering,

Johns Hopkins University, Baltimore MD 21218

Table S1. Gene expression datasets used in this study

Table S2: Ligand genes included in this study

Table S3: Receptor genes included in this study

Table S4: Means and standard deviations of gene expression

Table S5: Clinical trial results for bevacizumab by hormone receptor status

Table S6: Genes associated with VEGF- and semaphorin-based principal component PC3a

Table S7: Genes associated with VEGF- and semaphorin-based principal component PC4a

Figure S1: Relationship between PCA scores and triple negative status for tumor data set

Figure S2: Association of PCA scores with PAM50 subtypes

Figure S3: Heatmap of clusters based only on PC3a and PC4a

Figure S4: Relationship between PCA of all tumors and PCA of TNBC samples only

Figure S5: Relationship between PCA scores of overlapping samples from two TCGA datasets

Figure S6: Correlation of PCA loadings vectors between all tumor dataset and TCGA datasets

Figure S7: Gene expression of the MSL subtype

Figure S8: Consensus *K*-means clustering of all tumor samples

Figure S9: Consensus *K*-means clustering of TNBCs

Figure S10: Heatmaps of TCGA data

Figure S11: Survival analysis by clusters

Figure S12: Survival analysis based on PC4a and ESR1 expression

Figure S13: Association between PC4a score in the five PAM50 subtypes

Table S1: Gene expression datasets used in this study. As noted in the main text, samples must be untreated primary tumors. Unless otherwise noted, each sample in the datasets represents one tumor. The numbers of tumor samples are the actual number of samples used in the analysis; replicate samples were removed.

Dataset	N	Reference	Notes
GSE1456	159	65	
GSE1561	49	66	Core biopsy, >20% tumor cell content
GSE2034	286	67	Tumor cell content >70%, all lymph-node negative
GSE2603	99	68	Tumor cell content >70%
GSE2990	104	69	
GSE3494	251	70	
GSE5327	58	71	All ER-
GSE5847	28	72	47 stroma, 48 tumor (LCM); Surgical samples
GSE7390	198	73	
GSE11121	200	74	Tumor cell content >40%
GSE20194	42	75	Tumor cell content >70%, 30 replicates
GSE20271	116	76	
GSE20437	42	77	Normal breast tissue (no tumors)
GSE21217	11	78	Surgical samples
GSE22093	68	79	
GSE22597	74	53	
GSE23988	61	79	
GSE24185	103	80	
GSE25066	508	81	
GSE31519	67	82	
GSE32072	25	83	
GSE36772	100	N/A	
GSE36773	49	N/A	

Table S2: Ligand genes included in this study

Gene	Probe ID	Full Name	Interactions	Effects	References
VEGFA	210512_s_at<br>210513_s_at<br>211527_x_at<br>212171_x_at	Vascular Endothelial Growth Factor A	VEGFR1<br>VEGFR2<br>NRP1<br>NRP2	Promotes angiogenesis	3
VEGFB	203683_s_at	Vascular Endothelial Growth Factor B	VEGFR1<br>NRP1	Promotes angiogenesis, particularly in the heart / coronary artery	3
VEGFC	209946_at	Vascular Endothelial Growth Factor C	VEGFR2<br>VEGFR3<br>NRP1<br>NRP2	Promotes lymphangiogenesis	3
PGF	209652_s_at<br>215179_x_at	Placental Growth Factor	VEGFR1<br>NRP1	Promotes angiogenesis, potentially through VEGFR1-mediated recruitment of inflammatory cells	3
SEMA3A	206805_at	Semaphorin 3A	NRP1	Inhibits angiogenesis	23<br>30
SEMA3B	203070_at<br>203071_at	Semaphorin 3B	NRP1<br>NRP2	Inhibits angiogenesis but is inactivated when cleaved by furin proteases	31
SEMA3C	203788_s_at<br>203789_s_at	Semaphorin 3C	NRP1<br>NRP2	Unclear, possibly pro-angiogenic	32<br>33
SEMA3D	215324_at	Semaphorin 3D	NRP1<br>NRP2	Inhibits angiogenesis	30
SEMA3E	206941_x_at	Semaphorin 3E	PLXND1	Inhibits angiogenesis	30<br>34<br>35
SEMA3F	206832_s_at<br>209730_at<br>35666_at	Semaphorin 3F	NRP2	Inhibits angiogenesis; may be more potent after cleavage by a furin protease	23<br>30<br>36<br>37
SEMA3G	219689_at	Semaphorin 3G	NRP2	Inhibits angiogenesis	30<br>38
SEMA4A	219259_at	Semaphorin 4A	PLXND1	Inhibits angiogenesis	28
SEMA4C	219039_at<br>46665_at	Semaphorin 4C	PLXNB2	Unknown	N/A
SEMA4D	203528_at	Semaphorin 4D	PLXNB1<br>PLXNB2	Promotes angiogenesis	29<br>39
SEMA5A	205405_at<br>213169_at	Semaphorin 5A	PLXNB3	Promotes angiogenesis	40
SEMA6A	215028_at<br>220454_s_at	Semaphorin 6A	PLXNA2<br>PLXNA4	Soluble extracellular domain inhibits HUVEC migration	41
				Inhibition by a miRNA increases endothelial cell sprouting	42
SEMA6B	220778_x_at	Semaphorin 6B	PLXNA4	Silencing in HUVECs results in reduced response to VEGF and FGF	27
SEMA6D	N/A	Semaphorin 6D	PLXNA1	Possibly promotes angiogenesis (causes VEGFR2 phosphorylation in some cells)	43
SEMA7A	210083_at	Semaphorin 7A	PLXNC1	Induces corneal neovascularization	44

Table S3: Receptor genes included in this study

Gene	Probe ID	Full Name	Interactions
FLT1	204406_at<br>210287_s_at	VEGF Receptor 1	VEGFA<br>VEGFB<br>PGF
KDR	203934_at	VEGF Receptor 2	VEGFA<br>VEGFC
FLT4	210316_at	VEGF Receptor 3	VEGFC
NRP1	210510_s_at<br>210615_at<br>212298_at	Neuropilin 1	VEGFA<br>VEGFB<br>PlGF<br>SEMA3A<br>SEMA3B<br>SEMA3C<br>SEMA3D
NRP2	210841_s_at<br>210842_at<br>211844_s_at<br>214632_at	Neuropilin 2	VEGFA<br>VEGFC<br>SEMA3B<br>SEMA3C<br>SEMA3D<br>SEMA3F<br>SEMA3G
PLXNA1	221537_at<br>221538_s_at	Plexin A1	SEMA3*<br>SEMA6D
PLXNA2	207290_at<br>213030_s_at	Plexin A2	SEMA3*<br>SEMA6A
PLXNA3	203623_at	Plexin A3	SEMA3*
PLXNA4	N/A	Plexin A4	SEMA3*<br>SEMA6A<br>SEMA6B
PLXNB1	215668_s_at<br>215807_s_at	Plexin B1	SEMA4D
PLXNB2	208890_s_at<br>211472_at	Plexin B2	SEMA4C<br>SEMA4D
PLXNB3	205957_at	Plexin B3	SEMA5A
PLXNC1	206470_at<br>206471_s_at<br>213241_at	Plexin C1	SEMA7A
PLXND1	212235_at<br>38671_at	Plexin D1	SEMA3E<br>SEMA4A

* SEMA3 family members bind plexinA receptors after binding to neuropilins, but it is unclear exactly which plexinAs interact with which SEMA3s.

Table S4: Means and standard deviations of gene expression

Part A: Ligands

		Normal		All Tumors		TN Tumors		Non-TN Tumors	
**Gene**	**Probe**	**Mean**	**Std. Dev.**	**Mean**	**Std. Dev.**	**Mean**	**Std. Dev.**	**Mean**	**Std. Dev.**
VEGFA	210512_s_at	8.91	0.35	8.84	0.96	9.16	1.14	8.70	0.83
	210513_s_at	5.91	0.22	7.22	0.72	7.62	0.84	7.06	0.60
	211527_x_at	5.76	0.33	6.95	0.89	7.41	1.08	6.77	0.71
	212171_x_at	7.54	0.21	8.72	0.64	9.02	0.80	8.60	0.51
VEGFB	203683_s_at	5.63	0.36	6.52	0.51	6.44	0.54	6.55	0.50
VEGFC	209946_at	5.88	0.38	6.32	0.52	6.22	0.56	6.36	0.50
PGF	209652_s_at	6.07	0.46	5.92	0.38	5.97	0.44	5.90	0.35
	215179_x_at	9.96	0.40	8.91	0.60	8.90	0.70	8.91	0.56
SEMA3A	206805_at	4.85	0.23	5.16	0.33	5.26	0.37	5.11	0.30
SEMA3B	203070_at	5.75	0.24	5.91	0.29	5.97	0.28	5.89	0.29
	203071_at	6.38	0.62	6.74	0.76	6.30	0.43	6.92	0.79
SEMA3C	203788_s_at	6.00	0.40	5.76	0.67	5.42	0.50	5.90	0.68
	203789_s_at	9.41	0.52	8.29	1.39	7.13	1.36	8.76	1.09
SEMA3D	215324_at	3.90	0.13	3.90	0.14	3.91	0.14	3.89	0.14
SEMA3E	206941_x_at	4.06	0.24	3.80	0.23	3.72	0.17	3.83	0.24
SEMA3F	206832_s_at	4.36	0.26	4.85	0.43	4.64	0.31	4.94	0.44
	209730_at	6.89	0.37	6.96	0.46	6.73	0.42	7.05	0.44
	35666_at	8.57	0.45	8.53	0.61	8.00	0.46	8.74	0.52
SEMA3G	219689_at	7.18	0.86	6.58	0.66	6.38	0.70	6.66	0.62
SEMA4A	219259_at	8.02	0.30	8.16	0.38	8.25	0.39	8.12	0.36
SEMA4C	219039_at	8.10	0.30	8.02	0.35	8.04	0.34	8.02	0.36
	46665_at	9.79	0.35	9.01	0.51	9.00	0.51	9.01	0.52
SEMA4D	203528_at	7.35	0.37	7.42	0.60	7.67	0.68	7.31	0.53
SEMA5A	205405_at	7.85	0.61	6.89	0.49	6.81	0.45	6.93	0.50
	213169_at	8.95	0.80	6.90	0.64	6.71	0.62	6.98	0.64
SEMA6A	215028_at	6.59	0.88	4.10	0.57	4.03	0.43	4.13	0.61
	220454_s_at	5.92	0.27	6.62	0.39	6.71	0.38	6.59	0.39
SEMA6B	220778_x_at	6.89	0.28	7.02	0.29	7.03	0.30	7.02	0.29
SEMA7A	210083_at	6.09	0.20	6.32	0.37	6.37	0.38	6.29	0.36

Table S4: Means and standard deviations of gene expression

Part B: Receptors

		Normal		All Tumors		TN Tumors		Non-TN Tumors	
**Gene**	**Probe**	**Mean**	**Std. Dev.**	**Mean**	**Std. Dev.**	**Gene**	**Probe**	**Mean**	**Std. Dev.**
FLT1	204406_at	5.37	0.17	5.33	0.24	5.37	0.21	5.32	0.25
	210287_s_at	3.76	0.15	3.79	0.17	3.83	0.17	3.78	0.16
KDR	203934_at	6.23	0.60	6.02	0.49	5.94	0.50	6.05	0.48
FLT4	210316_at	4.43	0.21	4.35	0.21	4.38	0.24	4.34	0.20
NRP1	210510_s_at	5.52	0.28	6.18	0.53	6.30	0.63	6.13	0.48
	210615_at	4.16	0.18	4.47	0.25	4.52	0.26	4.45	0.24
	212298_at	6.09	0.77	6.48	1.00	6.41	1.08	6.51	0.97
NRP2	210841_s_at	6.40	0.19	6.81	0.28	6.92	0.29	6.76	0.26
	210842_at	4.78	0.24	4.85	0.34	4.94	0.36	4.82	0.32
	211844_s_at	4.43	0.16	4.72	0.25	4.82	0.33	4.68	0.20
	214632_at	4.59	0.18	4.99	0.41	5.17	0.55	4.91	0.31
PLXNA1	221537_at	6.95	0.40	7.04	0.30	7.16	0.30	6.98	0.27
	221538_s_at	7.67	0.80	7.23	0.66	7.45	0.73	7.14	0.60
PLXNA2	207290_at	4.80	0.16	4.97	0.30	5.03	0.31	4.94	0.29
	213030_s_at	5.54	0.24	6.13	0.54	6.26	0.62	6.08	0.50
PLXNA3	203623_at	6.68	0.24	6.94	0.47	6.92	0.47	6.95	0.48
PLXNB1	215668_s_at	6.68	0.27	6.89	0.37	6.93	0.37	6.88	0.37
	215807_s_at	6.66	0.48	7.24	0.68	6.99	0.59	7.34	0.68
PLXNB2	208890_s_at	8.74	0.99	9.24	0.68	9.05	0.71	9.32	0.66
	211472_at	5.86	0.22	5.92	0.30	5.97	0.32	5.90	0.29
PLXNB3	205957_at	6.18	0.28	6.46	0.39	6.54	0.40	6.43	0.38
PLXNC1	206470_at	5.37	0.15	5.86	0.48	5.91	0.52	5.84	0.46
	206471_s_at	4.64	0.46	5.11	0.40	5.15	0.42	5.09	0.38
	213241_at	6.78	0.67	7.17	0.90	7.01	0.96	7.23	0.88
PLXND1	212235_at	7.12	0.51	7.40	0.52	7.35	0.57	7.42	0.49
	38671_at	8.11	0.41	8.56	0.55	8.48	0.59	8.59	0.52

Table S5: Clinical trial results for bevacizumab by hormone receptor status

Trial	Response	Subgroup	Control Group	Avastin Group	Hazard Ratio	Reference
Phase 3 trial of paclitaxel plus bevacizumab in metastatic breast cancer	Median PFS (months)	ER-/PR-	4.6	8.8	0.53	12
		ER+/PR+	8	14.4	0.54	
Phase 3 trial of docetaxel plus bevacizumab in metastatic breast cancer	Median PFS (months)	ER-/PR-	N/A	N/A	0.68	52
		ER+/PR+	N/A	N/A	0.77	
Phase 3 trial of two types of chemotherapy plus bevacizumab in metastatic breast cancer	Median PFS (months)	Capecitabine ER-/PR-	4.2	6.1	0.70	53
		Capecitabine ER+/PR+	6.2	9.2	0.69	
	Median PFS (months)	Taxane + Anthracycline ER-/PR-	6.2	6.5	0.78	
		Taxane + Anthracycline ER+/PR+	8.2	10.3	0.61	
Neoadjuvant bevacizumab with chemotherapy	Pathological complete response rate (%)	ER-/PR-	47.1	51.5	N/A	54
		ER+/PR+	15.1	23.2	N/A	
Neoadjuvant bevacizumab with chemotherapy	Pathological complete response rate (%)	ER-/PR-	27.9	39.3	N/A	55
		ER+/PR+	7.8	7.7	N/A	

Table S6: Genes associated with VEGF- and semaphorin-based principal component 3a

Gene	Probe ID	Correlation coefficient		Gene	Probe ID	Correlation coefficient
APLNR	213592_at	0.6410		SEMA5A	213169_at	0.5313
SYDE1	44702_at	0.6204		ANGPTL2	213001_at	0.5307
SVEP1	213247_at	0.6169		ABCA6	217504_at	0.5303
IFFO1	209721_s_at	0.6074		SYT11	209197_at	0.5300
CD34	209543_s_at	0.5992		SEPT11	214293_at	0.5270
HEG1	212822_at	0.5879		ERG	213541_s_at	0.5260
CD93	202877_s_at	0.5850		PECAM1	208982_at	0.5247
NPR1	32625_at	0.5793		TOMM20	200662_s_at	-0.5252
ARHGEF15	205507_at	0.5773		ARL6IP1	211935_at	-0.5254
PDE2A	204134_at	0.5750		NHP2	209104_s_at	-0.5278
PCDH12	219656_at	0.5744		POLR2K	202635_s_at	-0.5287
FOLR2	204829_s_at	0.5730		TBCA	203667_at	-0.5292
GAS7	211067_s_at	0.5675		MYCBP	203360_s_at	-0.5317
ITIH5	219064_at	0.5648		CBX3	201091_s_at	-0.5329
MFNG	204153_s_at	0.5623		RBM35A	219121_s_at	-0.5338
FEZ1	203562_at	0.5621		NDUFB4	218226_s_at	-0.5350
STAB1	38487_at	0.5612		PAFAH1B3	203228_at	-0.5368
GJA4	204904_at	0.5562		TSEN34	218132_s_at	-0.5405
SELP	206049_at	0.5550		PAICS	201013_s_at	-0.5414
S1PR1	204642_at	0.5530		MIF	217871_s_at	-0.5428
JAM2	219213_at	0.5489		EPCAM	201839_s_at	-0.5451
ADAMTS2	214454_at	0.5470		ARF1	200065_s_at	-0.5469
GPR124	221814_at	0.5457		SNRPE	203316_s_at	-0.5472
LRP1	200785_s_at	0.5428		FKBP4	200894_s_at	-0.5495
NOTCH4	205247_at	0.5407		PRDX2	39729_at	-0.5559
RBMS3	206767_at	0.5406		RAB25	218186_at	-0.5566
FAT4	219427_at	0.5402		SRP9	201273_s_at	-0.5616
LUZP1	221832_s_at	0.5390		NDUFAB1	202077_at	-0.5671
CORO2B	209789_at	0.5376		PTGES3	200627_at	-0.5682
EHD2	221870_at	0.5353		TPD52	201689_s_at	-0.5771
MMP19	204575_s_at	0.5352		SPINT2	210715_s_at	-0.5815
F13A1	203305_at	0.5326		HSPE1	205133_s_at	-0.5905

Table S7: Genes associated with VEGF- and semaphorin-based principal component 4a

Gene	Probe ID	Correlation coefficient		Gene	Probe ID	Correlation coefficient
C14orf45	220173_at	0.6066		PLXNA1	221538_s_at	-0.5135
ESR1	202225_at	0.5999		AURKB	209464_at	-0.5144
CA12	203963_at	0.5997		SLC43A3	213113_s_at	-0.5145
GATA3	209603_at	0.5822		BOP1	212563_at	-0.5155
ERBB4	214053_at	0.5805		MICALL1	55081_at	-0.5160
PNPLA4	209603_at	0.5762		KIF2C	209408_at	-0.5162
FOXA1	204667_at	0.5707		VGLL1	215729_s_at	-0.5174
NME5	206197_at	0.5703		UBE2C	202954_at	-0.5189
AGR2	209173_at	0.5683		BUB1	209642_at	-0.5189
SCUBE2	219197_s_at	0.5667		MYBL2	201710_at	-0.5191
ABAT	209460_at	0.5636		RHBDF2	219202_at	-0.5191
PTGER3	213933_at	0.5633		TTK	204822_at	-0.5213
TBC1D9	212956_at	0.5624		FSCN1	201564_s_at	-0.5249
MLPH	218211_s_at	0.5613		COTL1	221059_s_at	-0.5256
DNAJC12	218976_at	0.5593		NCK2	203315_at	-0.5283
GOLSYN	218692_at	0.5576		TPX2	210052_s_at	-0.5319
NAT1	214440_at	0.5557		TMEM158	213338_at	-0.5332
GPD1L	212510_at	0.5420		PLOD1	200827_at	-0.5337
HEXIM1	202815_s_at	0.5359		CTPS	202613_at	-0.5337
COX16	217645_at	0.5299		PLOD3	202185_at	-0.5426
BCL2	203685_at	0.5244		SF3B3	200687_s_at	-0.5445
TFF1	205009_at	0.5234		GLT25D1	218473_s_at	-0.5457
MAPT	203929_s_at	0.5198		IRAK1	201587_s_at	-0.5544
PEX11A	205160_at	0.5183		EN1	220559_at	-0.5605
SEMA3C	203789_s_at	0.5157		HMGA1	206074_s_at	-0.5649
SLC22A5	205074_at	0.5143		FOXM1	202580_x_at	-0.5674
IL6ST	204863_s_at	0.5139		TTLL4	203702_s_at	-0.5711
MYB	204798_at	0.5127		CEBPB	212501_at	-0.5742
HNRPDL	209068_at	0.5119		MCM5	216237_s_at	-0.5758
CDKN2A	209644_x_at	-0.5116		CDC20	202870_s_at	-0.5900
MCAM	211042_x_at	-0.5125		SLC7A5	201195_s_at	-0.5963
MKI67	212022_s_at	-0.5133				

Figure S1.

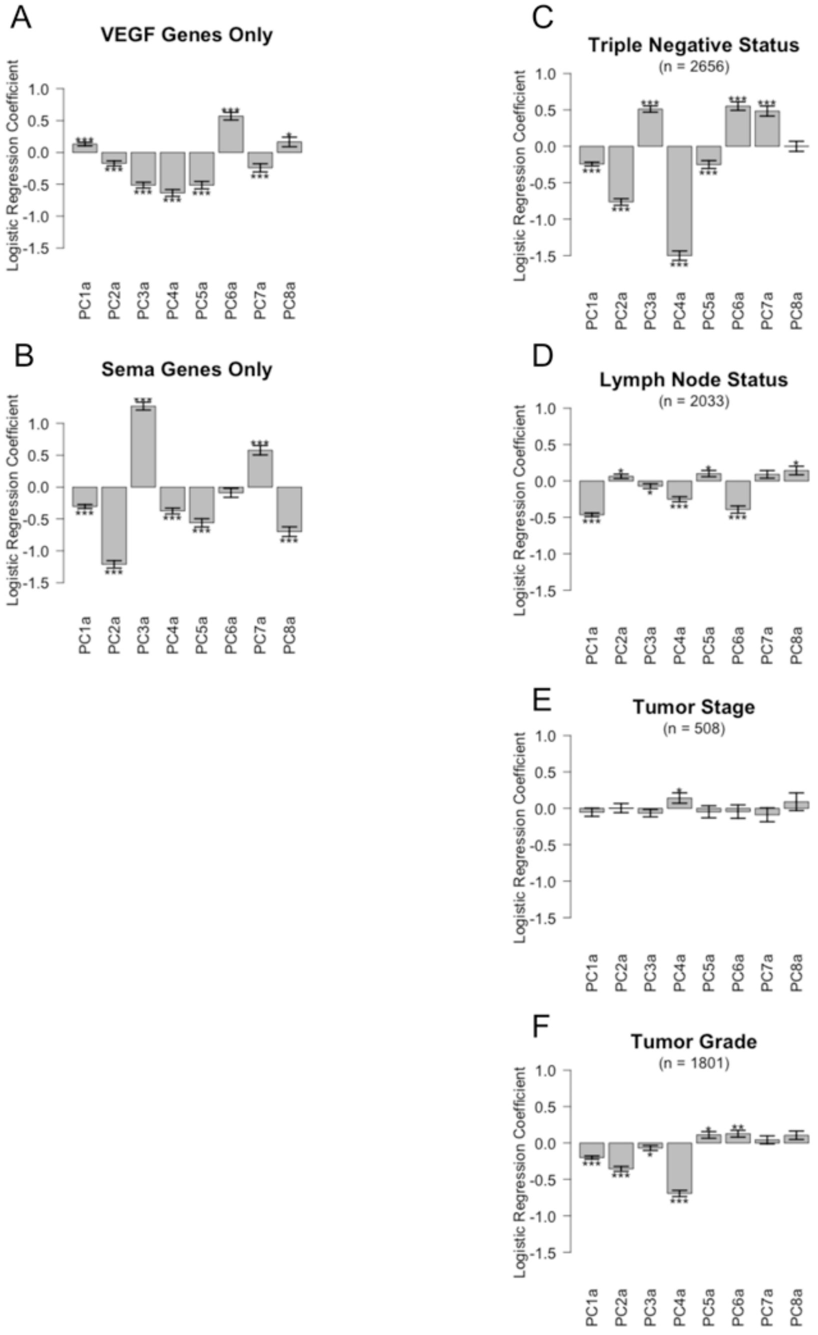

Figure S1: Relationship between principal component analysis (PCA) scores and triple negative status for tumor data set. A-B, Logistic regression coefficients for the first eight PCA scores for VEGF-related genes only (A) and for the semaphorin related genes only (B). The probe sets for NRP1 and NRP2 were included in both subsets of the data. **C-F**, Logistic regression coefficients for the combined VEGF/semaphorin geneset for triple negative status (C), lymph node status (D), tumor stage (E), and tumor grade (F). The value of n in C-F indicates how many samples had the relevant annotated data available.

Figure S2.

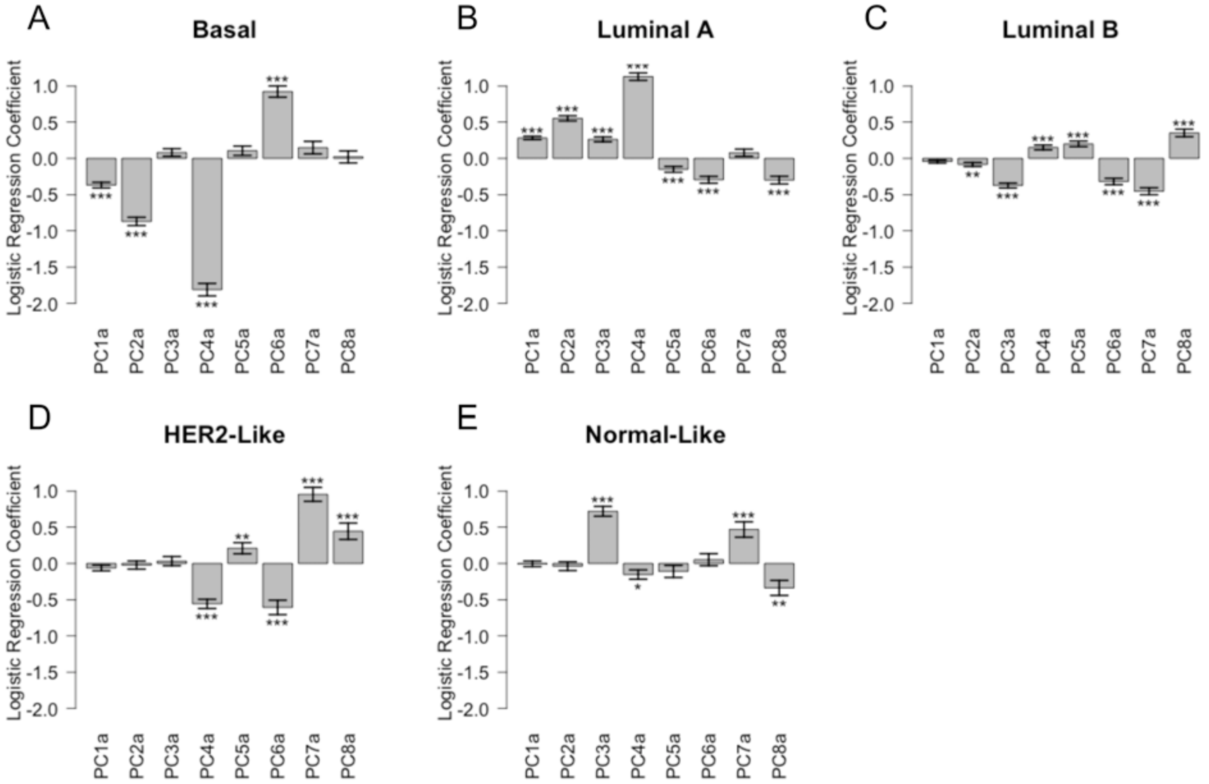

Figure S2: Association of PCA scores with PAM50 subtypes. Logistic regression coefficients for the first 8 PCs of the data set comprising all of the tumors. The largest association was between the 4th PC and the basal subtype (A). The 4th PC had a large inverse association with the luminal A subtype (B). The coefficients for the luminal B (C), HER2-like (D), and normal-like (E) subtypes were relatively small.

Figure S3.

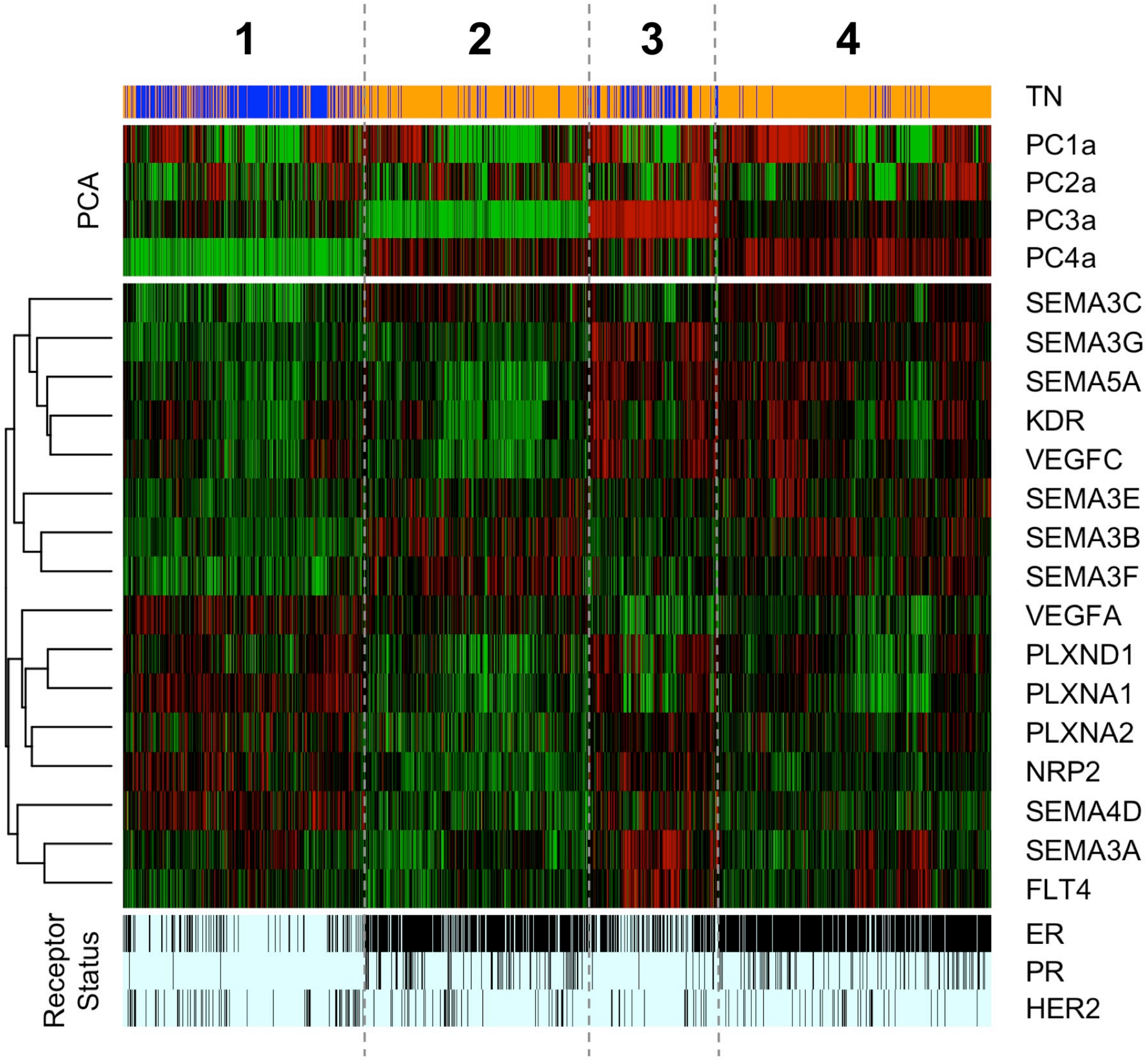

Figure S3: Heatmap of clusters based only on PC3a and PC4a. K-means cluster analysis of only the two principal components with high correlations with TN status (PC3a and PC4a) revealed two clusters with high TN content (1 and 3), and two with low prevalence of TNBC (2 and 4). Receptor status (light blue for negative, black for positive) for ER, PR, and HER2 showed that ER status was most associated with the VEGF/Sema gene expression.

The four clusters in the heatmap corresponded to:
(1) high VEGFA, SEMA4D, NRP2, PLXNA1, and low SEMA3B, SEMA3C, SEMA3E, SEMA3F, SEMA3G;
(2) high SEMA3B, SEMA3C, SEMA3F;
(3) high VEGFC, KDR, SEMA3G, SEMA5A and low VEGFA, SEMA3B, SEMA3C, SEMA3E, SEMA3F; and
(4) no consistent pattern of expression.
Most TNBC samples fell into the high VEGFA/SEMA4D cluster, with the high VEGFC/SEMA3G cluster containing the next highest amount of TNBC samples. Notably, both cluster 1 and cluster

3 demonstrated low expression of the anti-angiogenic genes SEMA3B, SEMA3C, SEMA3E, and SEMA3F. Among ER status, PR status, and HER2 status, ER and PR appeared to have a significant association with the clustering pattern, with ER-/PR-negative samples predominant in the high VEGFA/SEMA4D and high VEGFC/ NRP1/NRP2/PLXND1 clusters and ER-/PR-positive samples predominant in the other two clusters. This may indicate an important role for ER and PR in the transcription of the VEGF- and semaphorin-related genes considered here.

Figure S4.

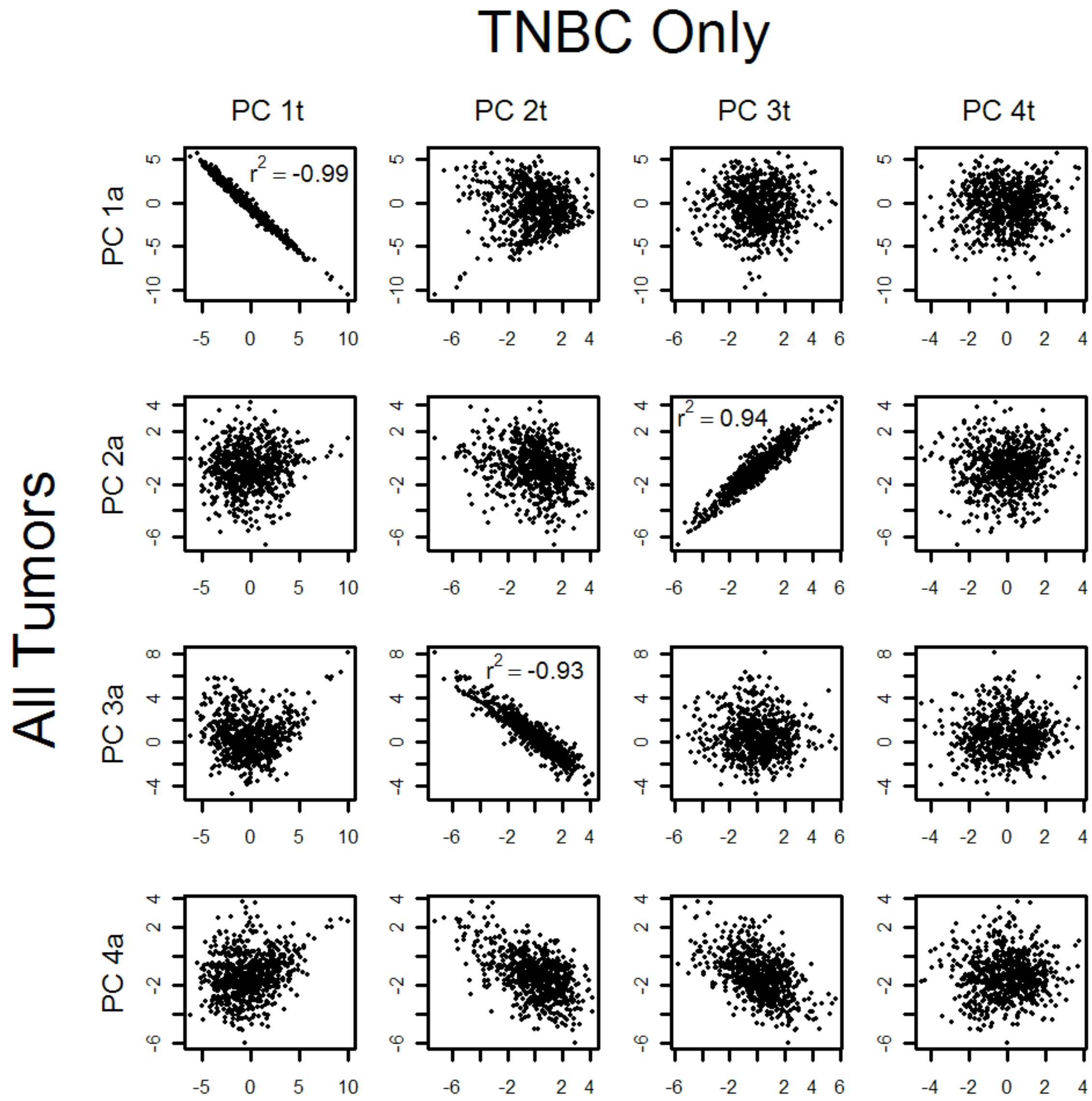

Figure S4: Relationship between PCA of all tumors and PCA of TNBC samples only. Scatterplots of the scores for TNBC samples from the all-tumor PCA and from the TNBC-only PCA reveal that PC1 is highly inversely correlated between the two analyses. Tumor PC2a is positively correlated with TNBC PC3t, while tumor PC3a is negatively correlated with TNBC PC2t. Tumor PC4a has no corresponding component in the TNBC PCA; this is due to the lack of variation of this component in the TNBC dataset (TNBC tumors typically score lowly on the 4th tumor PC).

Figure S5.

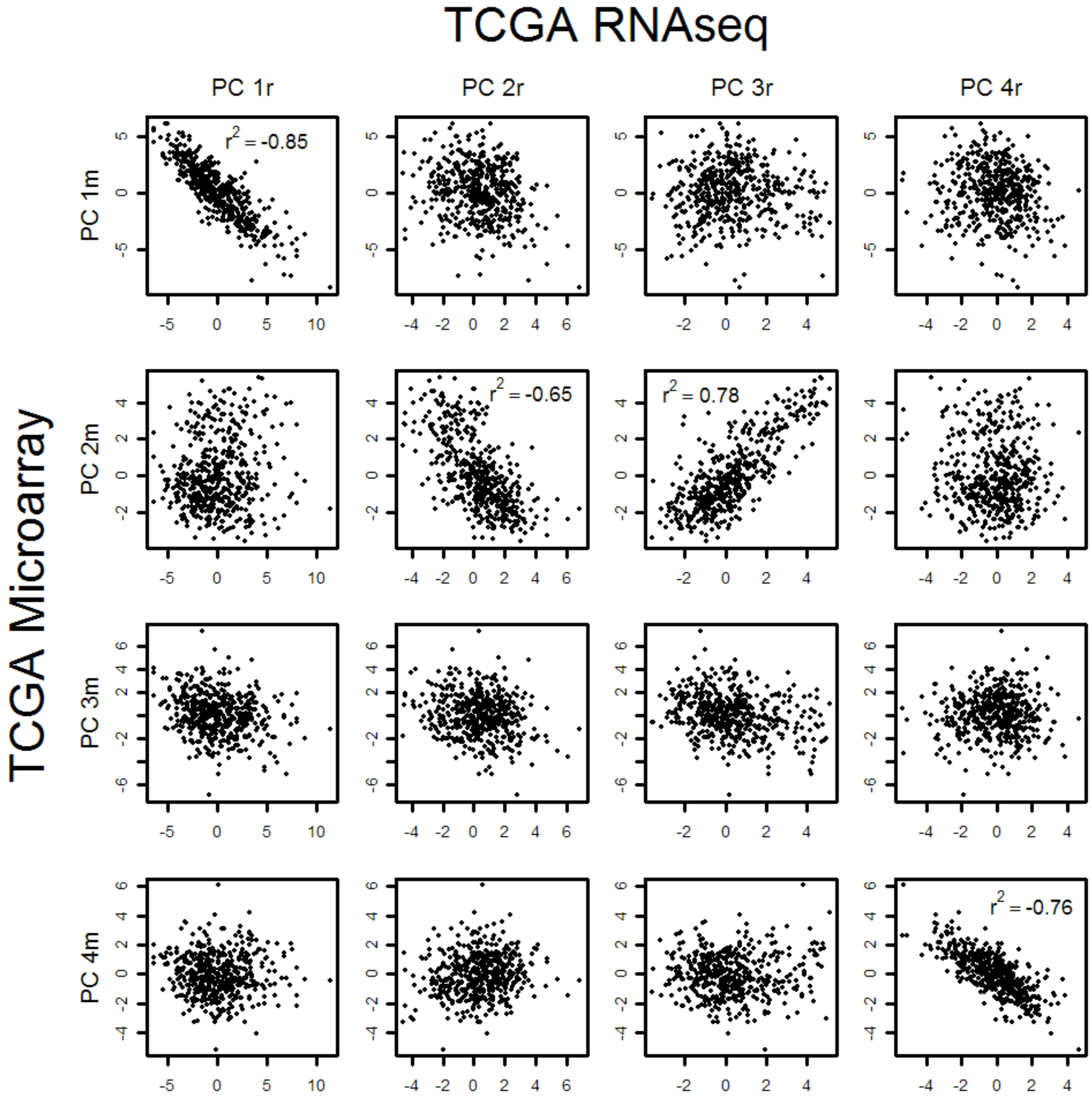

Figure S5: Relationship between PCA scores of overlapping samples from two TCGA datasets. Scatterplots of the scores for TCGA samples analyzed by microarray and by RNA-Seq show a strong correlation between the first component for each platform. The correlation of PC2m scores with both PC2r and PC3r scores is consistent with the relationships of these components with TN status (see figure 6).

Figure S6.

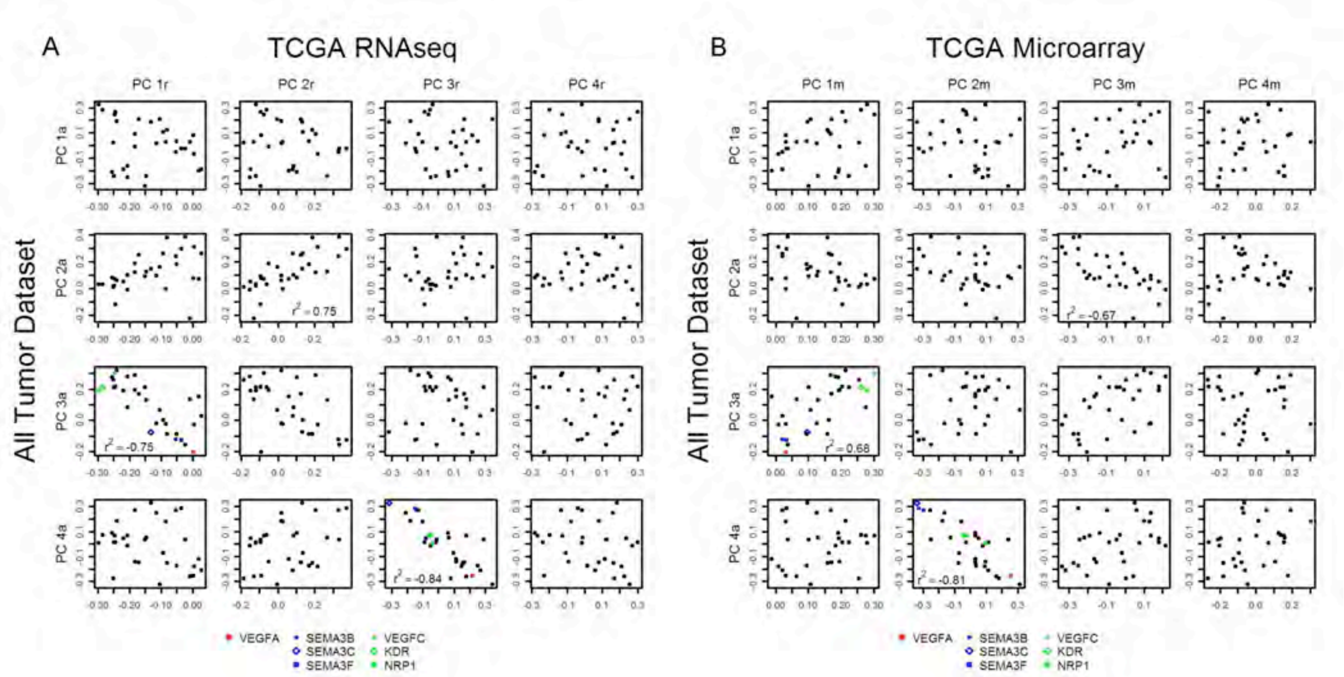

Figure S6: Correlation of PCA loadings vectors between all tumor dataset and TCGA datasets. A, Gene loadings between the 2,656-tumor GEO dataset and the TCGA RNA-Seq dataset showed weak correlations for several components. Importantly, patterns of gene expression were conserved across datasets/platforms: PC4a/PC3r had VEGFA expression and SEMA3B/3C/3F loading in opposite directions. **B,** Gene loadings between the 2,656-tumor GEO dataset and the TCGA microarray dataset also showed weak correlations for several components. In this case, PC4a and PC2t shared the VEGF/SEMA3 signature.

Figure S7.

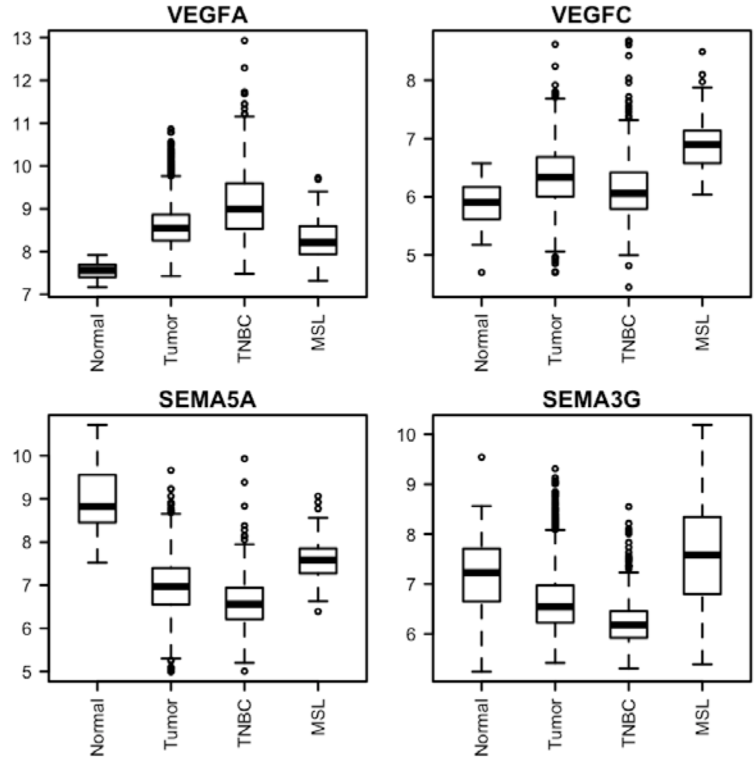

Figure S7: Gene expression of the MSL subtype. The MSL TNBC subtype had many genes whose expression resembled the all-tumor dataset more than the TNBC dataset, including VEGFA, SEMA5A, and SEMA3G. An exception to this was VEGFC, which had higher expression in the MSL subtype than in any other grouping.

Figure S8.

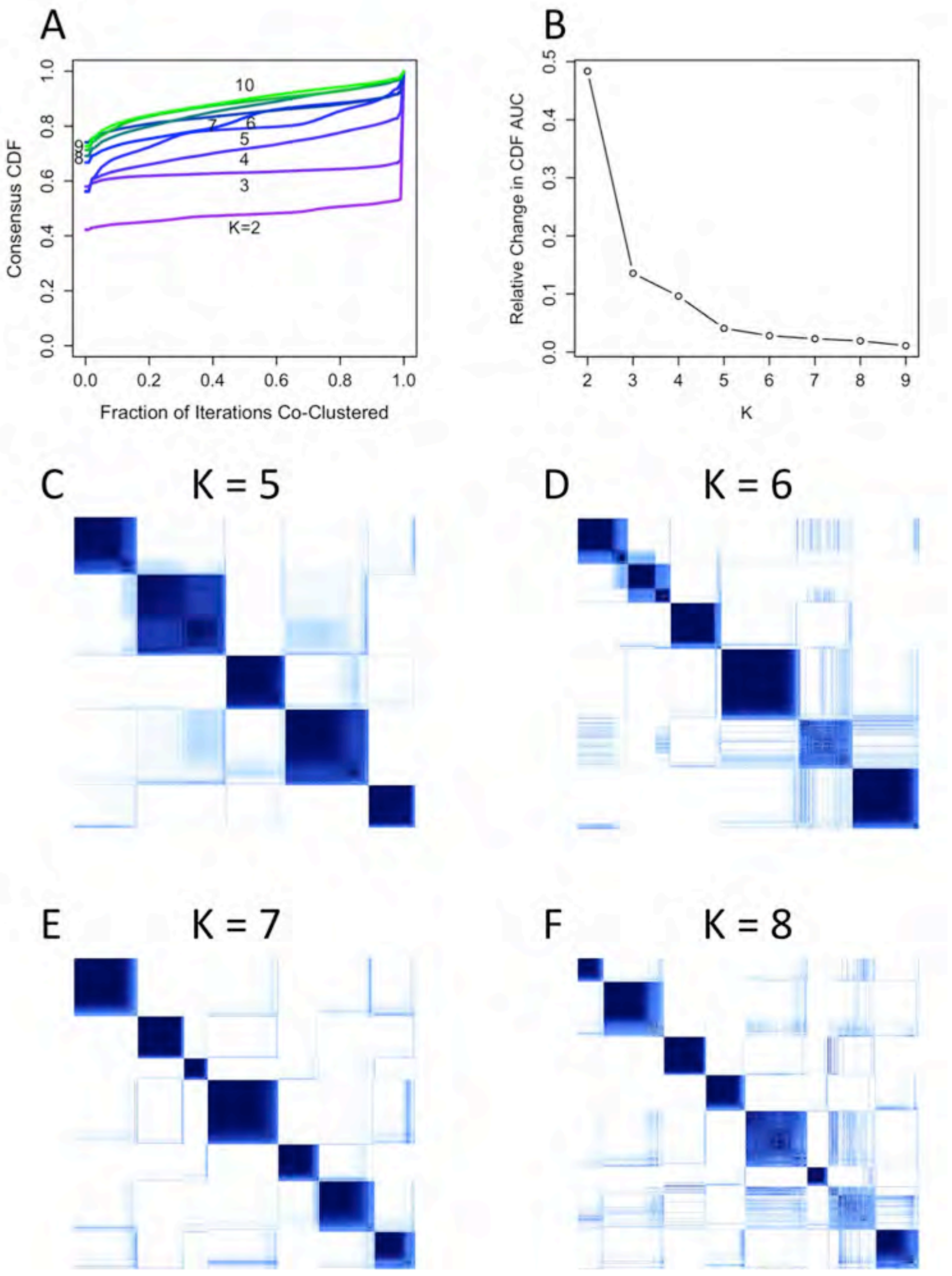

Figure S8: Consensus *K*-means clustering of all tumor samples. A, Cumulative consensus distribution curves showing the fraction of samples that co-clustered during 100 iterations of the *K*-means algorithm for all tumors. **B**, Relative change in area under the consensus CDF for K = 2 through 9. **C-F,** Consensus matrices for K = 5 through 8, with darker shades of blue indicating sample pairs that co-clustered more frequently.

Figure S9.

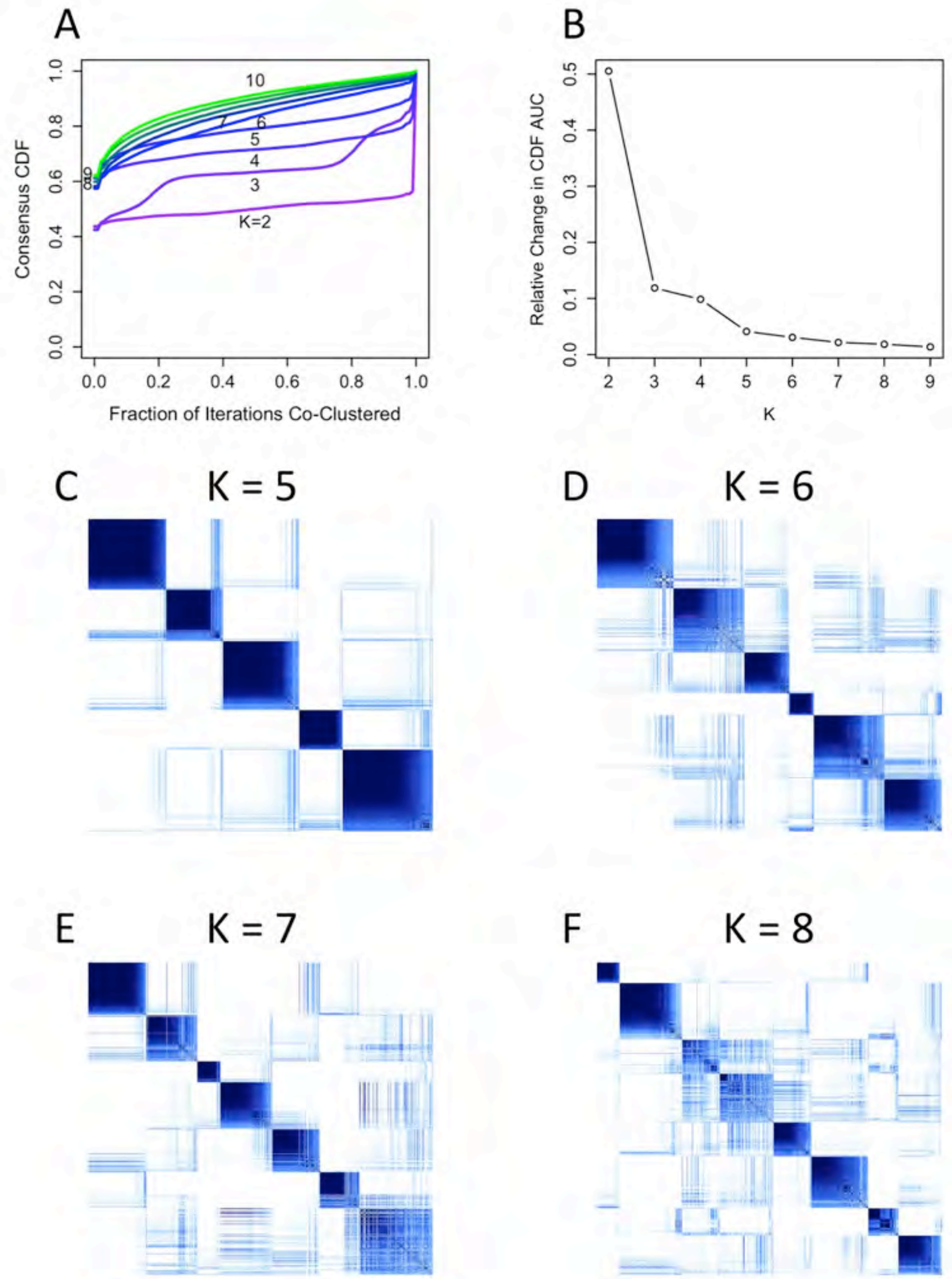

Figure S9: Consensus *K*-means clustering of TNBCs. A, Cumulative consensus distribution curves showing the fraction of samples that co-clustered during 100 iterations of the *K*-means algorithm for TNBC tumors. **B**, Relative change in area under the consensus CDF for K = 2 through 9. **C-F,** Consensus matrices for K = 5 through 8, with darker shades of blue indicating sample pairs that co-clustered more frequently.

Figure S10.

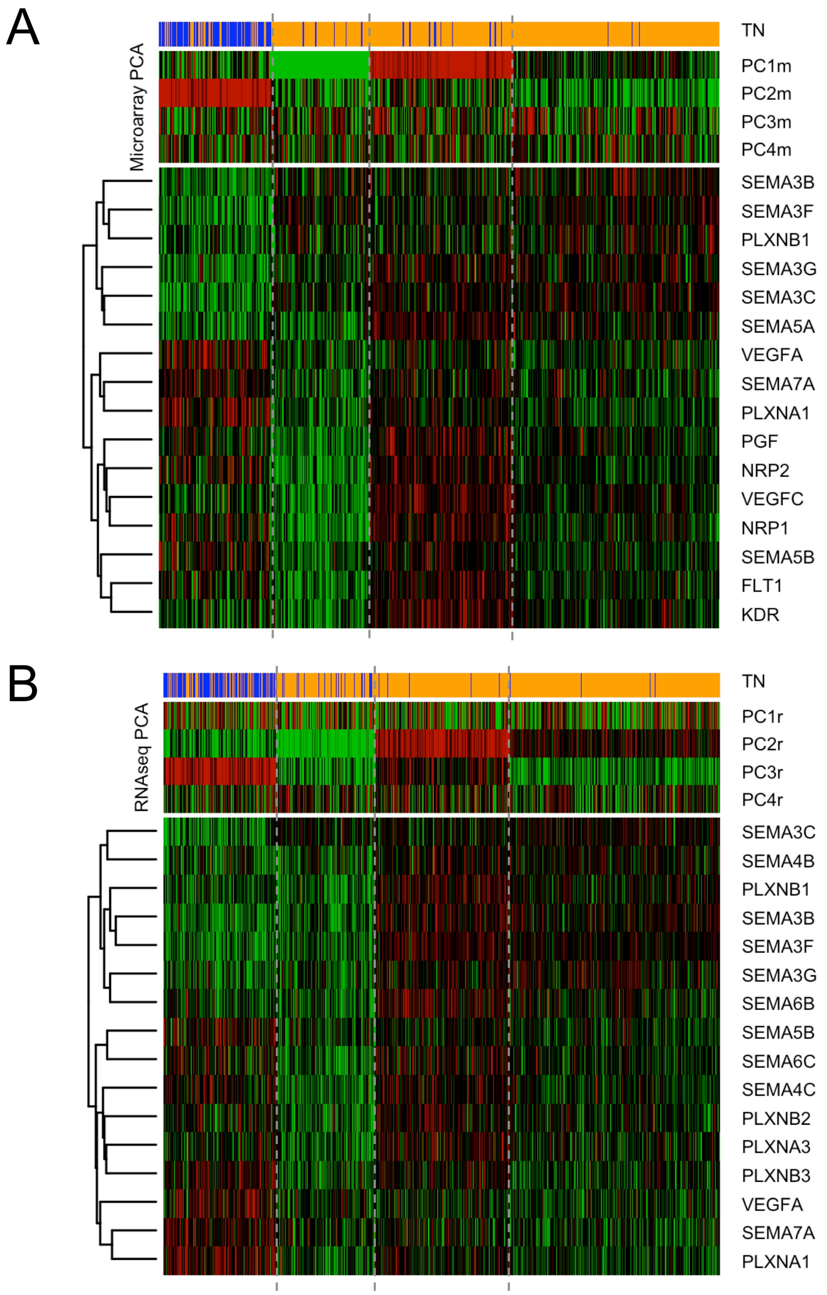

Figure S10: Heatmaps of TCGA data. A, Microarray data from Figures 6A and 6B were clustered based the 1st and 2nd principal component scores of the 537 samples (columns). The genes included here (rows) were those whose 1st and 2nd PC loadings vector had a magnitude greater than 0.24. **B,** RNAseq data from Figures 6C and 6D were clustered based on the 2nd and 3rd principal components of 750 samples. The genes included here were those whose 2nd and 3rd PC loadings vector had a magnitude greater than 0.23. In both heatmaps, red indicates high expression and green indicates low expression.

Figure S11.

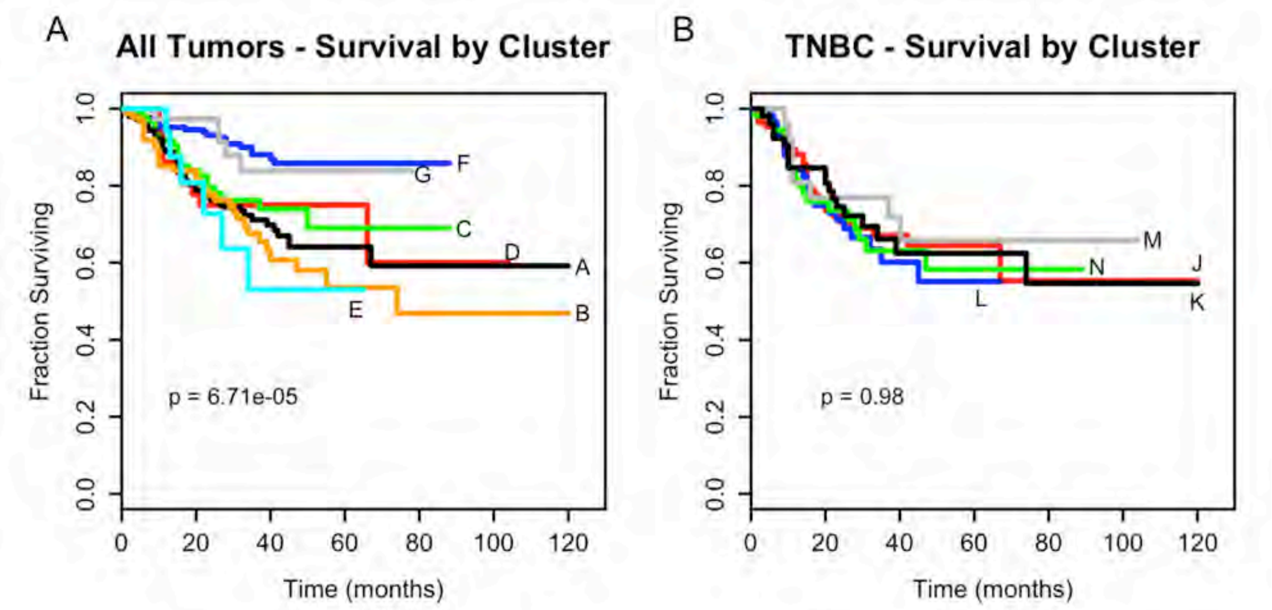

Figure S11: Survival analysis by clusters. A, Overall survival for the 7 VEGF-/Sema-based tumor clusters shows significant differences, particularly between clusters F and G with favorable prognoses and the remaining clusters. Cluster letters correspond to those in Figure 4. **B,** Overall survival for the 5 VEGF-/Sema-based TNBC clusters showed no significant differences in prognosis between clusters. Cluster numbers correspond to those in Figure 5.

Figure S12.

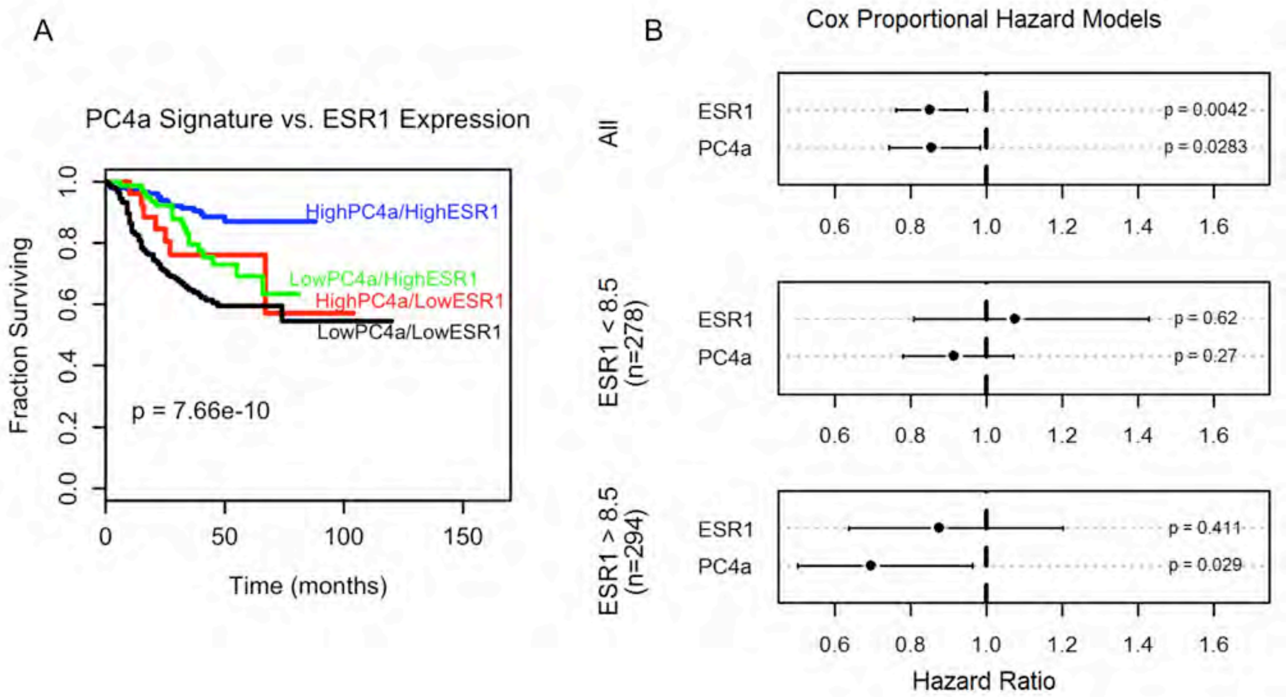

Figure S12: Survival analysis based on PC4a and ESR1 expression. A, Low PC4a scores and low ESR1 expression ("LowPC4a/LowESR1") were both associated with poorer prognoses. In high ESR1-expressing tumors ("HighESR1"), low PC4a scores resulted in poor prognosis as well**,** while high PC4a scores resulted in significantly better prognoses. **B,** Cox proportional hazard models reinforced the overall independence of ESR1 expression and PC4a score. Both factors were significantly associated with survival in a model of all tumors with survival data (top panel). In tumors with low ESR1 expression (middle panel), neither factor was significant. In a model of tumors with high ESR1 expression (bottom panel), PC4a score, but not ESR1 expression, had significant association with survival.

Figure S13.

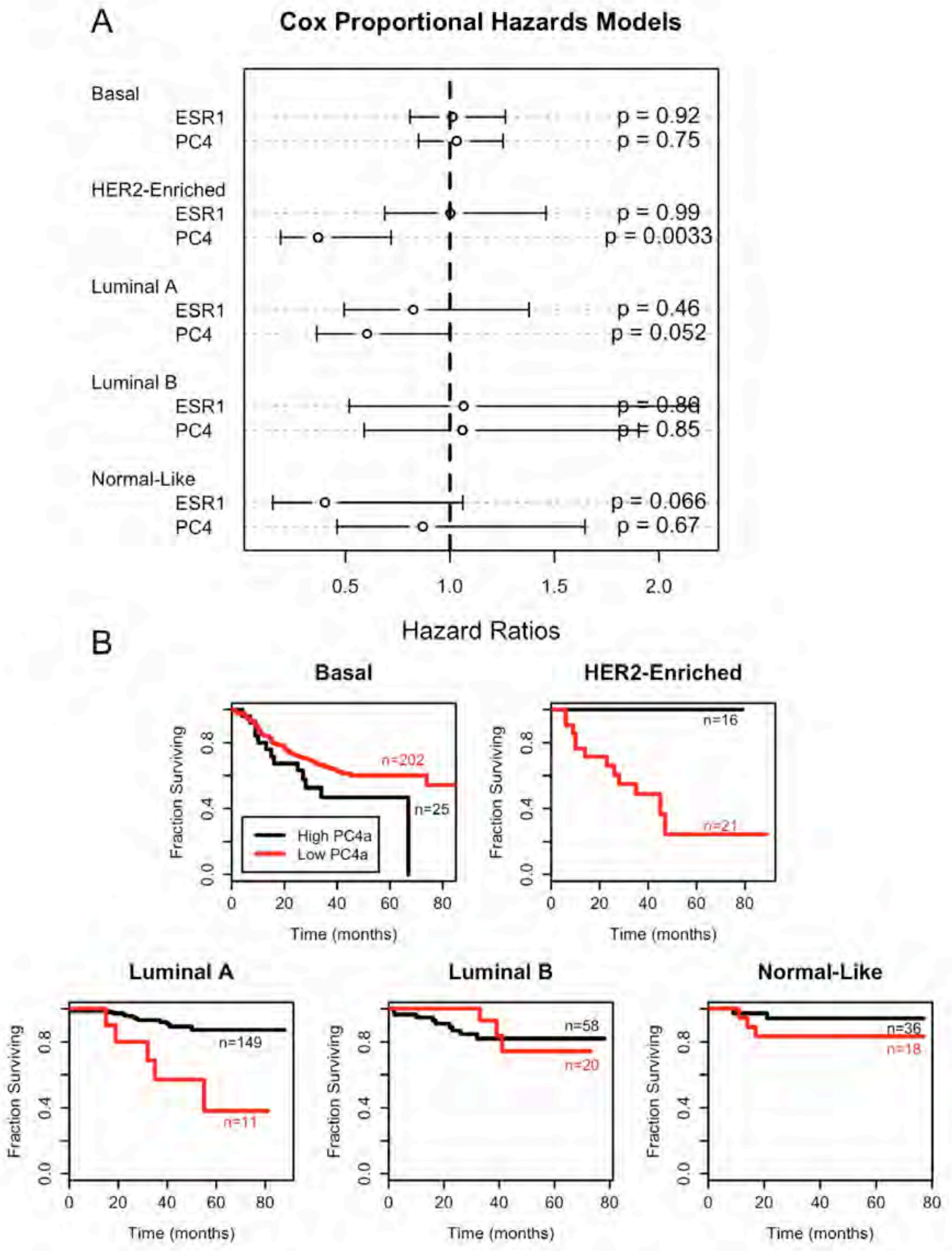

Figure S13: Association between PC4a score in the five PAM50 subtypes. A, Cox proportional hazard models for each PAM50 subtype demonstrated that PC4a score only was significantly associated with survival in the HER2-enriched subtype, while ESR1 expression was not significantly associated with survival in any of the subtypes. Hazard ratios indicate the effect of increasing PC4a score or ESR1 expression; thus, a lower hazard ratio indicates that high PC4a scores are associated with improved prognosis. PC4a scores and ESR1 expression were

included as continuous variables. **B,** PC4a scores below the median were typically associated with poorer prognoses except in the basal subtype. As the basal subtype is associated with low PC4a scores, very few samples in the basal subtype had high PC4a scores. This low number of samples explains the steep drop-off in the upper left plot.
